# Width Stability of Rotationally Symmetric Metrics

**DOI:** 10.1007/s12220-025-02020-5

**Published:** 2025-06-24

**Authors:** Hunter Stufflebeam, Paul Sweeney

**Affiliations:** 1 Department of Mathematics, University of Pennsylvania, Philadelphia, PA USA; 2 Dipartimento di Matematica, Università di Trento, via Sommarive 14, 38123 Povo di Trento, Italy

**Keywords:** Scalar curvature, Stability, Convergence of manifolds, 53C21 (Primary), 58J60, 53C24 (Secondary)

## Abstract

In 2018, Marques and Neves proposed a volume preserving intrinsic flat stability conjecture concerning their width rigidity theorem for the unit round 3-sphere. In this work, we establish the validity of this conjecture under the additional assumption of rotational symmetry. Furthermore, we obtain a rigidity theorem in dimensions at least three for rotationally symmetric manifolds, which is analogous to the width rigidity theorem of Marques and Neves. We also prove a volume preserving intrinsic flat stability result for this rigidity theorem. Lastly, we study variants of Marques and Neves’ stability conjecture. In the first, we show Gromov–Hausdorff convergence outside of certain “bad” sets. In the second, we assume non-negative Ricci curvature and show Gromov–Hausdorff stability.

## Introduction

A classical question in Riemannian geometry is how curvature controls the size and topology of a manifold. Typically, comparison and rigidity theorems exemplify this phenomenon. In [36], Marques and Neves proved such a theorem for Riemannian 3-spheres under the presence of a lower scalar curvature bound and the existence of a minimal surface produced via min–max methods. Precisely, they show that if there is a Riemannian metric on the 3-sphere with positive Ricci curvature and scalar curvature at least 6, then the Simon–Smith width, $W_g$, of the metric is at most $4\pi$. Moreover, equality is attained if and only if the metric is isometric to the standard unit round metric on the 3-sphere.

One can naturally wonder what happens when the hypotheses of a rigidity theorem are perturbed—if a geometric object almost satisfies the hypotheses of a rigidity theorem, is the object close to an object exemplifying the rigidity statement? These types of questions are typically phrased as “stability” problems. At the 2018 IAS Emerging Topics Workshop on *Scalar Curvature and Convergence* [50], Marques and Neves conjectured the following stability theorem related to their rigidity theorem above. But first, for notational convenience, we define $g_0^n$ to be the unit round metric on the *n*-sphere.

### Conjecture 1.1

Fix $D,V<\infty$. Suppose $(\mathbb {S}^3, g_k)$, $k=1,2,\ldots ,$ are Riemannian 3-spheres which satisfy

$$\begin{aligned} \textrm{Scal}_{g_k}\geqslant 6(1-k^{-1}), \textrm{MinA}_{g_k}\geqslant 4\pi (1-k^{-1}), {\textrm{diam}}_{g_k}\left( \mathbb {S}^3\right) \leqslant D, \text { and } \textrm{Vol}_{g_k}^3\left( \mathbb {S}^3\right) \leqslant V. \end{aligned}$$

Then $(\mathbb {S}^3, g_k)$ converges in the volume preserving intrinsic flat ($\mathcal{V}\mathcal{F}$) sense to the unit round sphere $(\mathbb {S}^3,g_0^{3})$.

In Conjecture 1.1, the original condition on the width is replaced with a stronger condition on $\textrm{MinA}$, which is defined for a general Riemannian *n*-manifold $\left( M^n,g\right)$ by

$$\begin{aligned} \textrm{MinA}_g:=\inf \left\{ \textrm{Vol}_g^{n-1}(\Sigma ):\Sigma \text {is a closed minimal hypersurface in}M\right\} . \end{aligned}$$

In fact, since the width $W_g$ is achieved by the area of a minimal surface, it is always true that $W_g\geqslant \textrm{MinA}_g$. Conjecture 1.1 also drops the assumption of $\textrm{Ric}>0$; in the proof of Marques–Neves’ rigidity theorem, $\textrm{Ric}>0$ is only used to ensure that the manifold contains no stable minimal 2-spheres. By Marques–Neves [36, Appendix A], we see that if the scalar curvature of a 3-manifold is sufficiently close to 6 and $\textrm{MinA}$ is sufficiently close to $4\pi$, then the manifold contains no stable minimal embedded surfaces. Lastly, the imposed bounds on diameter and volume guarantee, by Wenger’s compactness theorem [56], the existence of a subsequence, $\{(\mathbb {S}^3, g_{k_j})\}$, that converges in the Sormani–Wenger intrinsic flat ($\mathcal {F}$) topology [52] to an integral current space.

In this paper, we prove Conjecture 1.1 under the additional assumption of rotational symmetry, but without the assumption of a volume upper bound. We can even extend our results to all dimensions, $n\geqslant 3$. Our first result is the following.

### Theorem A

Fix $n\geqslant 3, \delta >0$, $D>0$. Let $({\mathbb {S}}^n,g)$ be a rotationally symmetric metric on the *n*-sphere. There exists an $\varepsilon =\varepsilon (n,D,\delta )>0$ such that if

- $$\textrm{diam}_g(\mathbb {S}^n)\leqslant D$$,
- $$\textrm{Scal}_g\geqslant n(n-1)(1-\varepsilon )^2$$,
- $\textrm{MinA}_g\geqslant \omega _{n-1}(1-\varepsilon )^{n-1}$, where $\omega _{n-1}$ is the volume of the standard unit round $(n-1)$-sphere,

then $$\textrm{d}_{\mathcal{V}\mathcal{F}}((\mathbb {S}^n, g), (\mathbb {S}^n, g_0^{n})):=\textrm{d}_{\mathcal {F}}((\mathbb {S}^n, g), (\mathbb {S}^n, g_0^{n}))+|\textrm{Vol}^n_g(\mathbb {S}^n)-\textrm{Vol}^n_{g_{0}^{n}}(\mathbb {S}^n)|\leqslant \delta $$.

We remark that without the uniform lower bounds on $\textrm{MinA}$ to prevent bubbling along the sequence as in Conjecture 1.1, counterexamples can be constructed as shown by the second author in [54]. These examples are rotationally symmetric; thus, even with our added hypothesis of rotational symmetry, the $\textrm{MinA}$ condition is necessary. We note that we do not need a volume bound for our proof because it ends up being a *conclusion* (see also Park–Tian–Wang [41, Remark 1.5]).

In dimension two, Máximo and the first author [40] proved a stability theorem which says that strictly convex 2-spheres, all of whose simple closed geodesics are close in length to $2\pi$, are $C^0$ Cheeger–Gromov close to the round sphere. In other words, Conjecture 1.1 is true in full generality in dimension 2, without any added symmetry assumptions and with a stronger notion of distance in the conclusion.^1^ In [4], Bamler and Máximo prove another version of Conjecture 1.1 without any symmetry assumption, but under the stronger curvature assumption that $\textrm{sec}>0$ and $\textrm{Scal}\geqslant 6$ instead of solely $\textrm{Scal}\geqslant 6$. Their result is also phrased in terms of the $C^0$ Cheeger–Gromov distance (which implies $\mathcal {F}$-convergence). It is interesting to note that despite the similarity of these results, the respective proof methods are quite different; the main techniques of [40] and the present work come from metric geometry and geometric measure theory, whereas the techniques of [4] come from Ricci flow.

Next, we state an extension to all dimensions of Marques–Neves’s width rigidity theorem where *rotational width*, $W^{\textrm{rot}}_g$, (see Definition 2.5) is used in place of the *Simon–Smith width*, $W_g$, in dimensions greater than three.

### Theorem B

Let $n\geqslant 3$ and *g* be a rotationally symmetric Riemannian metric on the n-sphere, ${\mathbb {S}}^n$ such that

- $$\textrm{Scal}_g\geqslant n(n-1)$$,
- $$W^n_g\geqslant \omega _{n-1}$$.

where $W^3_g = W_g$ and $W^n_g = W^{\textrm{rot}}_g$ for $n\geqslant 4$. Then $(\mathbb {S}^n, g)$ is isometric to $(\mathbb {S}^n, g_{0}^n)$.

Furthermore, it is interesting to compare the width rigidity of Marques–Neves with Min-Oo’s conjecture: if *g* is a smooth metric on the hemisphere $\mathbb {S}^n_+$ such that the scalar curvature satisfies $\textrm{Scal}_g\geqslant n(n-1)$, the induced metric on the boundary $\partial \mathbb {S}^n_+$ agrees with the standard unit round metric on ${\mathbb {S}}^{n-1}$, and the boundary $\partial \mathbb {S}^n_+$ is totally geodesic with respect to *g*, then *g* is isometric to the standard unit round metric on $\mathbb {S}^n_+$. Perhaps surprisingly, Min-Oo’s conjecture turned out to be false in general with $n\geqslant 3$ as seen by an example of Brendle, Marques, and Neves [8] (see also Corvino–Eichmair–Miao [17] and the work of the second author [55]). Nevertheless, many special cases of Min-Oo’s Conjecture are known to be true (Brendle–Marques [7], Hang–Wang [24, 25], and Hu–Liu–Shi [27]). In dimension $n=2$, Min-Oo’s conjecture is true and is contained in an old theorem of Toponogov (see [30] Theorem 3.4.10). In [53], the first author proved a stability version of this Min-Oo Conjecture/Theorem.

Other stability theorems involving scalar curvature and rotational symmetry have also been proven. Lee and Sormani investigated the intrinsic flat stability of the positive mass theorem [34] and the Penrose inequality [33] under the assumption of rotational symmetry. More recently, Park, Tian, and Wang [41, Theorem 1.3] proved that given $A,D>0$ and a sequence of oriented rotationally symmetric Riemannian 3-manifolds without boundary $(M_j^3,g_j)$ such that ${\textrm{diam}}_{g_j}\left( M_j\right) <D$, $\textrm{Scal}_{g_j}\geqslant 0$, and $\textrm{MinA}_{g_j}\geqslant A>0$, then there is a subsequence that converges to a metric space $(M_\infty ,g_\infty )$ such that $\textrm{Vol}^3_{g_{j_k}}(M_{j_k})$ converges to the mass $\textbf{M}(M_\infty )$, and where $g_\infty$ is a $C^0$, $H^1$, rotationally symmetric metric. Moreover, in a certain sense $g_\infty$ has non-negative generalized scalar curvature. Therefore, by this result, we already know that there is a subsequence in Conjecture 1.1 that $\mathcal {F}$-converges to such a limit space. The novel point of the conclusion of Theorem A is that the limit of such a sequence is a smooth Riemannian manifold, specifically, the unit round sphere. We do not, however, appeal to the compactness result [41, Theorem 1.3] in our arguments here.

If we add a Ricci non-negative hypothesis to Conjecture 1.1, then we can replace the $\textrm{MinA}_g$ lower bound with a lower bound on $W^n_g$ and prove the following measured Gromov–Hausdorff stability theorem for rotationally symmetric metrics.

### Theorem C

Fix $n\geqslant 3$ and $\delta >0$. There exists an $\varepsilon =\varepsilon (n, \delta )>0$ such that if *g* is a rotationally symmetric metric on the *n*-sphere $\mathbb {S}^n$ satisfying

- $$\textrm{Scal}_g\geqslant n(n-1)(1-\varepsilon )^2$$,
- $$\textrm{Ric}_g\geqslant 0$$,
- $$W^n_g\geqslant \omega _{n-1}(1-\varepsilon )^{n-1},$$

then $$\textrm{d}_{\textrm{mGH}}((\mathbb {S}^n, g), (\mathbb {S}^n, g_{0}^n)):=\textrm{d}_{\textrm{GH}}((\mathbb {S}^n, g), (\mathbb {S}^n, g_0^{n}))+|\textrm{Vol}^n_g(\mathbb {S}^n)-\textrm{Vol}^n_{g_{0}^{n}}(\mathbb {S}^n)|\leqslant \delta .$$

We also note that without the non-negative Ricci curvature assumption, the second author in [54] constructed a sequence of rotationally symmetric 3-manifolds with width at least $4\pi$ which are not $\textrm{GH}$-close to the unit round *n*-sphere. This example can be easily generalized to higher dimensions and $W^{\textrm{rot}}_g$.

Naturally, it is interesting to consider the relationship between Gromov–Hausdorff and intrinsic flat limits for sequences of manifolds when both types of limits exist. In general, suppose a sequence of Riemannian manifolds $M_k$ is convergent in both the Gromov–Hausdorff and intrinsic flat senses, with Gromov–Hausdorff limit $X_\infty$. From the convergence theory, it can be shown (see, e.g., Sormani–Wenger [51, Lemma 2.6]) that the integral current $T_\infty$ arising as the intrinsic flat limit of the $M_k$ has support $\textrm{spt}(T_\infty )\subset X_\infty$. Strict containment is possible if, for example, along the sequence “cancellation” occurs. In other words, intrinsic flat limits are generally isometrically embedded *subsets* of Gromov–Hausdorff limits when both exist. Matveev and Portegeis [39] show that, when the $M_k$ have a uniform lower bound on volume and Ricci curvature and a uniform upper bound on diameter, this containment is an equality—the Gromov–Hausdorff and intrinsic flat limits agree. In particular, Matveev–Portegeis’ work shows that we also get $\mathcal {F}$-closeness in Theorem C.

Lastly, as a result of the proofs of the above theorems we have a third version of the stability theorem in rotational symmetry—this time with respect to a convergence studied by Dong and Song in [20, Theorem 1.3] (see also Dong [19]) to resolve a conjecture of Huisken and Ilmanen [28, p. 430] about the stability of the positive mass theorem. Recently, a convergence of this type was also used by Bryden and Chen for stability theorems related to tori [9]. Roughly, the idea of this convergence is to remove a controlled “bad” set from each manifold in the sequence, so that what remains converges in the Gromov–Hausdorff sense to the desired limit. The notion of “bad” set varies slightly in the literature, but the unifying idea is that the “bad” set is geometrically small. In particular, we show

### Theorem D

Fix $n\geqslant 3$, $D>0$, and $\delta >0$. There exists an $\varepsilon =\varepsilon (n, \delta , D)>0$ such that if *g* is a rotationally symmetric metric on the *n*-sphere $\mathbb {S}^n$ satisfying

- $$\textrm{diam}_g(\mathbb {S}^n)\leqslant D$$;
- $$\textrm{Scal}_g\geqslant n(n-1)(1-\varepsilon )^2$$;
- $$\textrm{MinA}_g\geqslant \omega _{n-1}(1-\varepsilon )^{n-1},$$

then there exists a smooth domain $Z\subset \mathbb {S}^n$ with at most two connected components satisfying

$$\begin{aligned}\textrm{Vol}_g^n(Z)+\textrm{Vol}^{n-1}_g(\partial Z)\leqslant \delta ,\end{aligned}$$

so that $$\textrm{d}_{\textrm{GH}}((\mathbb {S}^n\setminus Z, g), (\mathbb {S}^n, g_{0}^n))\leqslant \delta $$.

The following is an outline of the paper. In Sect. 2, we provide the necessary background and preliminaries on rotationally symmetric manifolds, min–max theory, Gromov–Hausdorff convergence, and Sormani–Wenger intrinsic flat convergence. In Sect. 3, we prove Theorem B. In Sect. 4, we prove some preliminary propositions needed in the proof of the stability theorems. Finally, in Sect. 5, we prove Theorems A, C, and D.

### A Comment on Notation

Aside from other notation which we will introduce in the coming sections, we emphasize here that we follow tradition in using notation such as $\Psi =\Psi (x)=\Psi (x_1, x_2,\ldots : a_1,a_2,\ldots )$ to denote a non-negative function, which may change from line to line, depending on any number of variables $x_i$ and any number of parameters $a_i$ with the property that if the $a_i$ are all held fixed, $\Psi \searrow 0$ as $x_i\rightarrow 0$.

Throughout the paper, we will also denote the Hausdorff metric by $\textrm{d}_{\textrm{H}} (\cdot ,\cdot )$, the Gromov–Hausdorff ($\textrm{GH}$) distance by $\textrm{d}_{\textrm{GH}}(\cdot ,\cdot )$, the measured Gromov–Hausdorff ($\textrm{mGH}$) distance by $\textrm{d}_{\textrm{mGH}}(\cdot ,\cdot )$, the Sormani-Wenger intrinsic flat ($\mathcal {F}$) distance by $\textrm{d}_{\mathcal {F}}(\cdot ,\cdot )$, and the Volume Preserving intrinsic flat ($\mathcal{V}\mathcal{F}$) distance by $\textrm{d}_{\mathcal{V}\mathcal{F}}(\cdot ,\cdot )$.

## Background and Preliminaries

### Rotationally Symmetric Manifolds: An Overview

Since our main objects of interest in this paper are rotationally symmetric metrics on compact manifolds, we will work in coordinates that are well suited to this particular situation. Namely, we will view such an *n*-dimensional $(M^n,g)$ as a warped product of a line segment (0, *D*) with the round $(n-1)$-sphere, endowed with a metric

$$\begin{aligned} g=ds^2+f(s)^2g_{0}^{n-1} \end{aligned}$$

where $g_{0}^{n-1}$ is the round metric on $\mathbb {S}^{n-1}$. Here, *f*(*s*) is a smooth, non-negative function on (0, *D*). In particular, it is well known (see, e.g., [42]) that if $\lim _{x\rightarrow 0^+}f(x)=0$, then the smoothness of the metric implies that (after smoothly extending *f* to $s=0$):

- $$f'(0)=1$$ and $$f^{(even)}(0)=0$$;
- $$f(s)>0$$ if $$s\in (0,D)$$;
- if $\lim _{x\rightarrow D^-}f(x)=0$, then also $f'(D)=-1$ and $f^{(even)}(D)=0$.

We will be able to focus our attention in this paper on regions of $(M^n,g)$ where $f'(s)$ is nonzero and where a related coordinate system can also be used. In general, a rotationally symmetric manifold may be broken into parts based on the trichotomy

$$\begin{aligned} f'(s)< 0,f'(s)=0, \text { or } f'(s)>0, \end{aligned}$$

where $\{f'(s)=0\}$ contains the cylindrical pieces of the manifold. On a connected component with $f'(s)\ne 0$, we may instead use $r:=f(s)$ itself as the coordinate, and consider the metric

2.1 $$\begin{aligned} g=\frac{dr^2}{V(r)}+r^2g_0^{n-1} \end{aligned}$$

for a positive smooth function $V(r)=(f'(f^{-1}(r)))^2$. We note that *V*(*r*) satisfies its own appropriate derivative constraints at the endpoints of the interval, provided such a coordinate singularity point corresponds to a genuine smooth manifold point.

Focusing our attention now on such regions where both coordinate systems are available, we record the formulas we’ll need for the fundamental geometric objects of concern. Given a metric

2.2 $$\begin{aligned} g=\frac{dr^2}{V(r)}+r^2g_0^{n-1}=ds^2+f(s)^2g_0^{n-1}, \end{aligned}$$

we let $\Sigma _s:=\{s\}\times \mathbb {S}^{n-1}$ endowed with its induced metric. We then compute (see, e.g., [32] or [42]):

**Ricci Curvature:** 2.3 $$\begin{aligned} \textrm{Ric}_g(\partial _s,\partial _s)=-(n-1)\frac{f''(s)}{f(s)}. \end{aligned}$$
**Scalar Curvature:** 2.4 $$\begin{aligned} \begin{aligned} \textrm{Scal}_g&=(n-1)(n-2)\left( \frac{1-|f'(s)|^2}{f(s)^2}-\frac{2}{n-2}\frac{f''(s)}{f(s)}\right) \\&=\frac{n-1}{r^2}\left[ (n-2)(1-V(r))-rV'(r)\right] . \end{aligned} \end{aligned}$$
**Second Fundamental Form of**${\varvec{\Sigma _s}}$**:**
  2.5 $$\begin{aligned} \begin{aligned} A_{\Sigma _s}(X,Y)=g(\nabla _X \partial _s, Y)&=f(s)f'(s)\cdot g(X,Y)\\&=r\sqrt{V(r)}\cdot g(X,Y). \end{aligned} \end{aligned}$$
**Mean Curvature of**${\varvec{\Sigma _s}}$**:**
  2.6 $$\begin{aligned} \begin{aligned} H_g(\Sigma _s)=\textrm{tr}(A_{\Sigma _s})&=(n-1)\frac{f'(s)}{f(s)}\\&=(n-1)\frac{V(r)^{1/2}}{r}. \end{aligned} \end{aligned}$$
**Volume Form:** 2.7 $$\begin{aligned} d\textrm{Vol}^n_g=\frac{r^{n-1}}{V(r)^{1/2}}\textrm{d}\mathcal {L}^1(r)\otimes d\textrm{Vol}^{n-1}_{g_0^{n-1}}. \end{aligned}$$

We also record, for the reader’s convenience, that when $g=g_0^{n}$ is the unit round metric on an *n*-hemisphere, the coordinate representation in (2.2) has

2.8 $$\begin{aligned} {\left\{ \begin{array}{ll} V_0(r)=1-r^2 & \text { on }\quad [0,1] \\ f_0(s)=\sin (s) & \text { on }\quad [0,\pi /2]. \end{array}\right. } \end{aligned}$$

Lastly, we recall the following a priori estimate for $f'(s)$ when the scalar curvature is non-negative, whose proof is an application of the mean value theorem and the ODE for $\textrm{Scal}_g$:

#### Lemma 2.1

([41, Lemma 2.6]) If $\textrm{Scal}_g\geqslant 0$, then $|f'(s)|\leqslant 1$ everywhere. Consequently, $0\leqslant V(r)\leqslant 1$.

### Min–Max Theory

In this section, we introduce the main invariant of focus—the Simon–Smith min–max width $W_g$ of the metric *g* (and a high dimensional analog for rotationally symmetric metrics).

The modern min–max theory for constructing minimal surfaces in manifolds began in the work of Birkhoff and Lyusternik–Schnirelmann on the existence of simple closed geodesics in two-spheres [5, 35], and can be described succinctly as an extension of classical Morse theory to the area functional. Since the pioneering work of Almgren and Pitts in [2, 43, 44], min–max theory has been at the center of a veritable industry in geometric analysis, whose rich history is impossible to fully survey here. Amongst the many groundbreaking results in the area, we highlight the work of Marques–Neves [38], Irie–Marques–Neves [29], and Song [48] in the resolution of Yau’s conjecture [58] about the existence of infinitely many closed, embedded, minimal surfaces in every closed *n*-manifold ($3\leqslant n\leqslant 7$), and the work of Marques–Neves [37] in the resolution of the Willmore Conjecture.

We now turn to introducing the aspects of min–max theory relevant to us here—those of the *Simon–Smith* variant of the theory originally pioneered by Almgren–Pitts. This min–max theory, developed by Smith in [47] (see also Colding–De Lellis [16] for a fantastic accounting), produces a smooth and embedded minimal hypersurface in a Riemannian 3-manifold $$(M^3,g)$$. Consider a Riemannian 3-sphere $$(\mathbb {S}^3, g)$$. The starting point is the construction of *sweepouts*: one-parameter families of 2-spheres in $$(\mathbb {S}^3, g)$$, starting and ending at degenerate point spheres, which cover the whole of $$\mathbb {S}^3$$ in a topologically nontrivial way. The $$Simon--Smith width $$ of $$(\mathbb {S}^3, g)$$ is defined to be the infimum over the areas of largest 2-spheres in all such one-parameter family of 2-spheres “sweeping out” $$(\mathbb {S}^3, g)$$. Let us give the following more precise definition (cf. [16, 36]):

#### Definition 2.2

Given the standard embedding of $\mathbb {S}^3\hookrightarrow \mathbb {R}^4$, consider the level sets ${\overline{\Sigma }}_t:=(x^4)^{-1}(t)$, $t\in [-1,1]$, of the coordinate function $x^4:\mathbb {S}^3\subset \mathbb {R}^4\rightarrow \mathbb {R}$ (more generally, one could directly work with the level sets of a given Morse function on $(\mathbb {S}^3,g)$). Let ${\overline{\Lambda }}$ be the collection of all families $\left\{ \Sigma _t\right\} _{t\in [-1,1]}$ with the property that $\Sigma _t=F_t({\overline{\Sigma }}_t)$ for some smooth one-parameter family of diffeomorphisms $F_t:\mathbb {S}^3\rightarrow \mathbb {S}^3$ which are all smoothly isotopic to the identity $\textrm{id}:\mathbb {S}^3\rightarrow \mathbb {S}^3$. The *Simon–Smith width* of $(\mathbb {S}^3, g)$ is then defined to be the number

$$\begin{aligned}W_g:=\inf _{\left\{ \Sigma _t\right\} \in {\overline{\Lambda }}}\left\{ \sup _{t\in [-1,1]}\textrm{Vol}^2_g\left( \Sigma _t\right) \right\} .\end{aligned}$$

An important theorem in the Simon–Smith theory, which motivated our work in this paper, is the following result. In it, the area of the minimal sphere produced by min–max theory realizing the value $W_g$ can be viewed as a *size invariant* of $(\mathbb {S}^3, g)$. For other examples of size invariant, consider the lengths of closed geodesics which indicate the size and shape of positively curved two-spheres (see, e.g., Croke [18], Chap. 3 of [30], or [40]).

#### Theorem 2.3

(Marques–Neves [36]) Let $(\mathbb {S}^3, g)$ have $\textrm{Ric}(g)>0$ and $\textrm{Scal}_g\geqslant 6$. Then there exists an embedded two-sided minimal surface $\Sigma$, diffeomorphic to ${\mathbb {S}}^2$, with Morse index one^2^ such that

$$\begin{aligned}W_g=\textrm{Vol}^2_g(\Sigma )\leqslant 4\pi ,\end{aligned}$$

with equality if and only if $(\mathbb {S}^3, g)$ is isometric to the unit round sphere $(\mathbb {S}^3, g_0^{n-1})$.

The collection ${\overline{\Lambda }}$ is commonly referred to as the *saturated family of sweepouts generated by *$\left\{ {\overline{\Sigma }}_t\right\}$; in general, given any fixed one-parameter family of surfaces such as $\left\{ {\overline{\Sigma }}_t\right\}$ in Definition 2.2, one can consider the collection of all one-parameter families $\left\{ \Sigma _t\right\}$ obtained by deforming the starting family along all such families of diffeomorphisms (this is the notion of *generation*, while *saturation* refers to the property of the collection being closed under all such deformations). In the rotationally symmetric case, it is easy to see that the specific collection $\overline{\Lambda }$ in Definition 2.2 is also generated by the following sweepout, which we term the *canonical sweepout*. It will be the focus of essentially all of our computations in the sequel.

#### Definition 2.4

(The Canonical Sweepout) Let

$$\begin{aligned} g=ds^2+f(s)^2g_{0}^{n-1} \text { with }s\in [0,D] \end{aligned}$$

be a smooth, rotationally symmetric metric on the three-sphere $\mathbb {S}^n$. Let $p_-$ be the “south pole” of the suspension, where $s=0$. The canonical sweepout is the one-parameter family $\{\Sigma _s\}_{s\in [0, D]}$ of two spheres, where $\Sigma _s:=(\textrm{dist}_{p_-})^{-1}(s)$. Each $\Sigma _s$ has the induced metric $f(s)^2g_0^{n-1}$, and therefore $\textrm{Vol}^{n-1}_g(\Sigma _s)=4\pi f(s)^{n-1}$.

Our theorems are stated for all dimensions $n\geqslant 3$; however, in high dimensions $n\geqslant 4$ *there is no Simon–Smith theory available*. In part, this is due to the fact that the regularity theory for minimal hypersurfaces produced from isotopy classes, which forms a key part of the Simon–Smith method, cannot prevent the presence of large singular sets, even in dimensions $4\leqslant n\leqslant 7$ (see White [57]). In particular, there seems to be *no known higher dimensional analog of Theorem* 2.3. Nonetheless, the rotational symmetry hypothesis allows us to avoid these issues. To state our theorems for $n\geqslant 4$, we consider both the closely related invariant $\textrm{MinA}$ and a higher dimensional width analog.

#### Definition 2.5

(*Rotational Width*) Let $n\geqslant 3$, and let

$$\begin{aligned} g=ds^2+f(s)^2g_{0}^{n-1} \text { with }s\in [0,D] \end{aligned}$$

be a smooth, rotationally symmetric metric on the *n*-sphere $\mathbb {S}^n$. Let $\{\Sigma _s\}_{s\in [0, D]}$ and define

$$\begin{aligned} W^{\textrm{rot}}_g:=\max _{s\in [0,D]}\textrm{Vol}^{n-1}_g(\Sigma _s). \end{aligned}$$

#### Remark 2.6

We note that in dimension three we have that $W^{\textrm{rot}}_g\ge W_g$, where strict inequality is possible. Consider for example a rotationally symmetric ellipsoid isometrically embedded in Euclidean $\mathbb {R}^4$ where the semi-axial length along the axis of rotation is arbitrarily small and the other three are of unit length, say $E_L:=\{x^2+y^2+z^2+w^2/L^2=1\}$ with $0<L\ll 1$. For all $L>0$, $W^{\textrm{rot}}_g=\textrm{Area}(E_L\cap \{w=0\})=4\pi$, whereas as $L\searrow 0$

$$\begin{aligned}W_g=\textrm{Area}(E_L\cap \{z=0\})=2\pi \left( 1+\frac{L^{2}}{\sqrt{1-L^2}}\textrm{arctanh}\left( \sqrt{1-L^2}\right) \right) \searrow 2\pi .\end{aligned}$$

We will state and prove our main results using this clearly weaker invariant (in particular, we have not *infimized* over any collection of sweepouts) because the largest leaf in the canonical sweepout strongly controls the global geometry of a rotationally symmetric $(\mathbb {S}^n, g)$ with the appropriate curvature bounds, as we will demonstrate. The other invariant, called $\textrm{MinA}$, is a familiar quantity related to stability problems in the context of the Sormani–Wenger Intrinsic convergence (see Sect. 2.4) and is defined as follows:

#### Definition 2.7

(*MinA*) Let $(M^n,g)$ be a Riemannian *n*-manifold. Then

$$\begin{aligned}\textrm{MinA}_g:=\inf \left\{ \textrm{Vol}_g^{n-1}(\Sigma ): \Sigma \text { is a closed minimal hypersurface in }M\right\} .\end{aligned}$$

Clearly, this is the *strongest* size invariant of the three that we have introduced, in the sense that a lower bound on $\textrm{MinA}_g$ gives a lower bound on $W_g$ in dimension 3 and $W^{\textrm{rot}}_g$. This invariant, originally introduced by Sormani in [49], was defined to control *bubbling* along sequences of manifolds converging in the intrinsic flat sense.

We end this subsection with a lemma which we will find useful in the proof of our main theorem. A general rotationally symmetric metric on the sphere may have many minimal hypersurfaces that could be either stable or unstable. However, if the metric has a lower bound on scalar curvature close to $n(n-1)$ and a lower bound on $\textrm{MinA}$ close to $\omega _{n-1}$, then the canonical sweepout contains exactly one minimal surface.

#### Lemma 2.8

Let $n\geqslant 3$. Then there exists an $c(n)>0$ such that for any $0<\varepsilon <c(n)$ if

$$\begin{aligned}g=ds^2+f(s)^2g_0^{n-1} \text { with }s\in [0,D]\end{aligned}$$

is a smooth, rotationally symmetric metric on the *n*-sphere $\mathbb {S}^n$ satisfying

- $$\textrm{Scal}_g\geqslant n(n-1)(1-\varepsilon )^2$$;
- $$\textrm{MinA}_g\geqslant \omega _{n-1}(1-\varepsilon )^{n-1}$$,

then the canonical sweepout $\{\Sigma _s\}_{s\in [0, D]}$ of $(\mathbb {S}^n,g)$ contains exactly one minimal hypersurface, which is necessarily unstable.

#### Proof

Since $f(0)=f(D)=0$, we can take an $s_0\in (0,D)$ such that $f(s_0)=\max _{s\in [0,D]} f(s)$. Then $f'(s_0)=0$ and so $\Sigma _{s_0}$ is a minimal sphere. Therefore, by using the assumption on $\textrm{MinA}_g$, we have $f(s_0)\geqslant (1-\varepsilon )$. Furthermore, by maximality, $f''(s_0)\leqslant 0$. For the sake of contradiction, suppose that there were another minimal sphere, say $\Sigma _{t_0}$ where without loss of generality $s_0<t_0<D$. We will obtain a contradiction by showing the existence of a stable $\Sigma _{r_0}$ of large area, which is impossible because of the lower scalar curvature bound (see Marques–Neves [36, Proposition A.1]). Indeed, if $\Sigma _{r_0}$ is any stable minimal sphere in the sweepout, then as is well known by using the Schoen–Yau rearrangement trick [46] in the stability inequality we see

2.9 $$\begin{aligned} \int _{\Sigma _{r_0}} \textrm{Scal}_{\Sigma _{r_0}} -|A_{\Sigma _{r_0}}|^2 \geqslant \int _{\Sigma _{r_0}} \textrm{Scal}_M. \end{aligned}$$

Applying the scalar curvature lower bound and using (2.9), we obtain that

2.10 $$\begin{aligned} \frac{(n-1)(n-2)}{f(r_0)^2}\textrm{Vol}^{n-1}_g(\Sigma _{r_0})=\int _{\Sigma _{r_0}}\textrm{Scal}_{\Sigma _{r_0}}\geqslant n(n-1)(1-\varepsilon )^2\textrm{Vol}^{n-1}_g(\Sigma _{r_0}).\nonumber \\ \end{aligned}$$

By rearranging (2.10), we obtain the following upper bound on the radius of $\Sigma _{r_0}$:

2.11 $$\begin{aligned} f(r_0)\leqslant \frac{1}{1-\varepsilon }\sqrt{\frac{n-2}{n}}. \end{aligned}$$

Therefore, if $\textrm{MinA}_g\geqslant \omega _{n-1}(1-\varepsilon )^{n-1}$ where $\varepsilon <c(n):=1-\left( \frac{n-2}{n}\right) ^\frac{1}{4}$, then $\{\Sigma _s\}_{s\in [0,D]}$ contains no stable minimal surfaces because if not the following implies contradiction when combined with (2.11):

$$\begin{aligned} 1-\varepsilon <\left( \frac{\textrm{MinA}_g}{\omega _{n-1}}\right) ^\frac{1}{n-1}\leqslant f(r_0). \end{aligned}$$

Thus, for $\textrm{MinA}_g$ so large it suffices to find a stable minimal $\Sigma _{r_0}$ to obtain a contradiction. If either of $\Sigma _{s_0}$, $\Sigma _{t_0}$ is stable then we are done, and otherwise we have $f'(s_0)=f'(t_0)=0$ with $f''(s_0), f''(t_0)<0$. Then *f* attains a local minimum at some $r_0$ in the interior of $[s_0, t_0]$ where $f'(r_0)=0$ and $f''(r_0)\geqslant 0$, so that $\Sigma _{r_0}$ is a stable minimal sphere and we may again conclude. $\square$

#### Remark 2.9

We note that as a result of the proof of Lemma 2.8, we know that $$f'(s)>0$$ on $$[0,s_0)$$ and $$f'(s_0)=0$$, where $$\Sigma _{s_0}$$ is the (only) minimal hypersurface in the canonical sweepout $$\{\Sigma _s\}_{s\in [0, D]}$$ of $$(\mathbb {S}^n,g)$$.

### Gromov–Hausdorff Convergence

Here we will review the Gromov–Hausdorff ($\textrm{GH}$) distance between two metric spaces. We refer the reader to Gromov [22] and Burago–Burago–Ivanov [10] for further details.

The Gromov–Hausdorff distance between two compact metric spaces $(X_1,d_1)$ and $(X_2,d_2)$ can be defined by

$$\begin{aligned} \textrm{d}_{\textrm{GH}}((X_1,d_1),(X_2,d_2))=\inf _{Z} \{\textrm{d}_{\textrm{H}}^Z(\phi _1(X_1),\phi _2(X_2))\} \end{aligned}$$

where the infimum is taken over all complete metric spaces $(Z,d^Z)$ and all distance preserving maps $\phi _i:X_i\rightarrow Z$, and where $\textrm{d}_{\textrm{H}}^Z$ denotes the standard Hausdorff distance between two compact subsets of $(Z,d^Z)$: For any compact $Y_1, Y_2\subset Z$,

$$\begin{aligned} \textrm{d}_{\mathrm {\textrm{H}}}^Z(Y_1,Y_2)=\inf \{r>0: Y_1\subset T_r(Y_2)\text { and } Y_2\subset T_r(Y_1)\} \end{aligned}$$

with $T_r(Y)= \{y\in Z: d_Z(y,Y)< r\}$. We say that a metric spaces $(X_j,d_j)$ converge in the $\textrm{GH}$-sense to a metric space $(X_\infty ,d_\infty )$ if

$$\begin{aligned} \textrm{d}_{\textrm{GH}}((X_j,d_j),(X_\infty ,d_\infty )) \rightarrow 0. \end{aligned}$$

Let (*M*, *g*) and (*N*, *h*) be Riemannian manifolds and $d_g$ and $d_h$ be the induced distance functions, respectively. If $d_{\textrm{GH}}((M,d_g),(N,d_h))=0$ then there is a Riemannian isometry from (*M*, *g*) to (*N*, *h*). Therefore, $\textrm{GH}$-distance defines a distance between two Riemannian manifolds.

### Sormani–Wenger Intrinsic Flat Convergence

In this section, we will review Sormani–Wenger intrinsic flat distance between two integral current spaces. Sormani and Wenger [52] defined intrinsic flat distance, which generalizes the notion of flat distance for currents in Euclidean space. To do so they used Ambrosio and Kirchheim’s [3] generalization of Federer and Fleming’s [21] integral currents to metric spaces. We refer the reader to [3] for further details about currents in arbitrary metric spaces and to [52] for further details about integral current spaces and intrinsic flat distance.

Let $(Z,d^Z)$ be a complete metric space. Denote by $\textrm{Lip}(Z)$ and $\textrm{Lip}_b(Z)$ the set of real-valued Lipschitz functions on *Z* and the set of bounded real-valued Lipschitz functions on *Z*, respectively.

#### Definition 2.10

([3, *Definition* 3.1]) We say a multilinear functional

$$\begin{aligned} T:\textrm{Lip}_b(Z)\times [\textrm{Lip}(Z)]^m\rightarrow {\mathbb {R}}\end{aligned}$$

on a complete metric space (*Z*, *d*) is an *m*-dimensional current if it satisfies the following properties.

(i) Locality: $T(f,\pi _1,\ldots ,\pi _m)=0$ if there exists an *i* such that $\pi _i$ is constant on a neighborhood of $\{f\ne 0\}$.
(ii) Continuity: *T* is continuous with respect to pointwise convergence of $\pi _i$ such that $\textrm{Lip}(\pi _i)\le 1$.
(iii) Finite mass: there exists a finite Borel measure $\mu$ on *X* such that
  2.12 $$\begin{aligned} |T(f,\pi _1,\ldots ,\pi _m)|\le \prod _{i=1}^m \textrm{Lip}(\pi _i) \int _Z |f| d\mu \end{aligned}$$
  for any $$(f,\pi _1,\ldots ,\pi _m)$$.

Ambrosio and Kirchheim call the minimal measure satisfying (2.12) the mass measure of *T* and denote it ||*T*||. We can now define many concepts related to a current. $\textbf{M}(T)=||T||(Z)$ is defined to be the mass of *T* and the canonical set of a *m*-current *T* on *Z* is

$$\begin{aligned} \text {set}(T)=\left\{ p\in Z \Big | \liminf _{r\rightarrow 0} \frac{||T||(B(p,r))}{r^m}>0\right\} . \end{aligned}$$

The support of *T* is

$$\begin{aligned} \text {spt}(T):= \textrm{spt} ||T|| = \left\{ z\in Z:||T||\left( B_z\left( r\right) \right)>0\quad \forall r >0\right\} . \end{aligned}$$

Ambrosio and Kirchheim proved that the closure of $\text {set}(T)$ is $\text {spt}(T)$. The boundary of a current *T* is defined as $\partial T:\textrm{Lip}_b(X)\times [\textrm{Lip}(X)]^{m-1}\rightarrow {\mathbb {R}}$, where

$$\begin{aligned} \partial T(f,\pi _1,\ldots ,\pi _{m-1})=T(1,f,\pi _1,\ldots ,\pi _{m-1}). \end{aligned}$$

Given a Lipschitz map $\phi :Z\rightarrow Z'$, we can pushforward a current *T* on *Z* to a current $\phi _\# T$ on $Z'$ by defining

$$\begin{aligned} \phi _{\#} T(f,\pi _1,\ldots ,\pi _m) = T(f\circ \phi ,\pi _1\circ \phi ,\ldots ,\pi _m\circ \phi ). \end{aligned}$$

A standard example of an *m*-current on *Z* is given by

$$\begin{aligned} \phi _\#\llbracket \theta \rrbracket (f,\pi _1,\ldots ,\pi _m) =\int _A (\theta \circ \phi ) (f\circ \phi )d(\pi _1\circ \phi )\wedge \cdots \wedge d(\pi _m\circ \phi ), \end{aligned}$$

where $\phi :A\subseteq {\mathbb {R}}^m\rightarrow Z$ is bi-Lipschitz and $\theta \in L^1(A,\mathbb {Z})$. We say that an *m*-current on *Z* is integer rectifiable if there is a countable collection of bi-Lipschitz maps $\phi _i: A_i\rightarrow X$ where $A_i\subseteq {\mathbb {R}}^m$ are precompact Borel measurable with pairwise disjoint images and weight functions $\theta _i\in L^1(A_i,\mathbb {Z})$ such that

$$\begin{aligned} T=\sum _{i=1}^\infty \phi _{i\#} \llbracket \theta _i\rrbracket . \end{aligned}$$

Moreover, we say an integer rectifiable current whose boundary is also integer rectifiable is an integral current. We denote the space of integral *m*-currents on *Z* as $\textbf{I}_m(Z)$. We say that the triple (*X*, *d*, *T*) is an *m*-dimensional integral current space if (*X*, *d*) is a metric space, $T\in \textbf{I}_m(\bar{X})$ where $\bar{X}$ is the metric completion of *X*, and $\text {set}(T)=X$. The next example ([52], cf. Allen–Bryden [1, Example 2.1]) explicitly shows how a closed oriented Riemannian manifold can be viewed as an integral current space.

#### Example 2.11

Let $(M^n,g)$ be a closed oriented Riemannian manifold. Then there is a naturally associated *n*-dimensional integral current space $\left( M,d_g,\llbracket M\rrbracket \right)$, where $d_g$ is the distance function induced by the metric *g* and $\llbracket M \rrbracket : \textrm{Lip}_b\left( M\right) \times \left[ \textrm{Lip}\left( M\right) \right] ^{n}\rightarrow {\mathbb {R}}$ is given by

$$\begin{aligned} \llbracket M \rrbracket =\sum _{i,j} \psi _{i\#} \llbracket \mathbbm {1}_{A_{ij}} \rrbracket \end{aligned}$$

where we have chosen a smooth locally finite atlas $\left\{ \left( U_i,\psi _i\right) \right\} _{i\in \mathbb {N}}$ of *M* consisting of positively oriented biLipschitz charts, $\psi _i: U_i\subseteq {\mathbb {R}}^n\rightarrow M$ and $A_{ij}$ are precompact Borel sets such that $\psi _i\left( A_{ij}\right)$ have disjoint images for all *i*, *j* and cover *M* $\mathscr {H}^n$ almost everywhere. Moreover, we have $\left| \left| \llbracket M \rrbracket \right| \right| =d\textrm{Vol}^n_g$.

The flat distance between two integral currents $T_1$, $T_2\in \textbf{I}(Z)$ is

$$\begin{aligned} \textrm{d}^Z_\textrm{F}(T_1,T_2) = \inf \{\textbf{M}(U)+\textbf{M}(V)\mid U\in \textbf{I}_m(X), V\in \textbf{I}_{m+1}(X), T_2-T_1=U+\partial V\}. \end{aligned}$$

The intrinsic flat $(\mathcal {F})$ distance between two integral current spaces $(X_1,d_1,T_1)$ and $(X_2,d_2,T_2)$ is

$$\begin{aligned} \textrm{d}_\mathcal {F}((X_1,d_1,T_1),(X_2,d_2,T_2)) = \inf _Z\{\textrm{d}_{\textrm{F}}^Z(\phi _{1\#}T_1,\phi _{2\#}T_2)\}, \end{aligned}$$

where the infimum is taken over all complete metric spaces $(Z,d^Z)$ and isometric embeddings $\phi _1:(\bar{X}_1,d_1)\rightarrow (Z,d^Z)$ and $\phi _2:(\bar{X}_2,d_2)\rightarrow (Z,d^Z)$. We note that if $(X_1,d_1,T_1)$ and $(X_2,d_2,T_2)$ are precompact integral current spaces such that

$$\begin{aligned} \textrm{d}_\mathcal {F}((X_1,d_1,T_1),(X_2,d_2,T_2))=0 \end{aligned}$$

then there is a current preserving isometry between $(X_1,d_1,T_1)$ and $(X_2,d_2,T_2)$, i.e., there exists an isometry $f:X_1\rightarrow X_2$ whose extension $\bar{f}:\bar{X}_1\rightarrow \bar{X}_2$ pushes forward the current: $\bar{f}_\#T_1=T_2$. We say a sequence of $(X_j,d_j,T_j)$ precompact integral current spaces converges to $(X_\infty ,d_\infty ,T_\infty )$ in the $\mathcal {F}$-sense if

$$\begin{aligned} \textrm{d}_\mathcal {F}((X_j,d_j,T_j),(X_\infty ,d_\infty ,T_\infty ))\rightarrow 0. \end{aligned}$$

If, in addition, $\textbf{M}(T_i)\rightarrow \textbf{M}(T_\infty )$, then we say $(X_j,d_j,T_j)$ converges to $(X_\infty ,d_\infty ,T_\infty )$ in the volume preserving intrinsic flat $(\mathcal{V}\mathcal{F})$ sense.

In this paper, our main tool for estimating the Gromov–Hausdorff and intrinsic flat distance between two spaces is the following key result of Lakzian and Sormani in [31]. In it, two manifolds are found to be close in one of these distances if there are large diffeomorphic subregions situated similarly in each space which are themselves close in the $C^0$ Cheeger–Gromov sense (defined in the following). Specifically, they proved the following:

#### Theorem 2.12

(Lakzian–Sormani [31]) Suppose that $M_1=(M, g_1)$ and $M_2=(M,g_2)$ are oriented precompact Riemannian manifolds with diffeomorphic subregions $U_i\subset M_i$ and diffeomorphisms $\psi _i:U\rightarrow U_i$ such that

$$\begin{aligned} \frac{1}{(1+\varepsilon )^2}\psi _2^*g_2(v,v)\leqslant \psi _1^*g_1(v,v)\leqslant (1+\varepsilon )^2\psi _2^*g_2(v,v) \end{aligned}$$

for every $v\in TU$ (i.e., $g_1$ and $g_2$ are close in the $C^0$ Cheeger–Gromov sense). We define the following quantities:

- $$D_{U_i}=\sup \{\textrm{diam}_{M_i}(W): \hbox { W is a connected component of}\ U_i\}$$;
- $$a>\pi ^{-1}\arccos \left( (1+\varepsilon )^{-1}\right) \max \{D_{U_1}, D_{U_2}\}$$;
- $$\lambda =\sup _{x,y\in U} |d_{M_1}(\psi _1(x),\psi _1(y))-d_{M_2}(\psi _2(x),\psi _2(y))|$$;
- $$h=\sqrt{ \lambda (\max \{D_{U_1}, D_{U_2}\}+\lambda /4) }$$;
- $$\overline{h}=\max \{h, \sqrt{\varepsilon ^2+2\varepsilon }D_{U_1},\sqrt{\varepsilon ^2+2\varepsilon }D_{U_2}\}$$.

Then the Gromov–Hausdorff distance between the metric completions $\overline{M_i}$ of the $M_i$ is bounded:

$$\begin{aligned} \textrm{d}_{\textrm{GH}}(\overline{M_1}, \overline{M_2})\leqslant a+2{\overline{h}}+\max \left\{ \textrm{d}_{\textrm{H}}^{M_1}(U_1, M_1), \textrm{d}_\textrm{H}^{M_2}(U_2, M_2)\right\} . \end{aligned}$$

Similarly, the intrinsic flat distance between $M'_i=\textrm{set}(\llbracket M \rrbracket )$ is bounded:

$$\begin{aligned} \textrm{d}_{\mathcal {F}}(M_1', M_2')&\leqslant \left( a+2{\overline{h}}\right) \left( \textrm{Vol}^n_{g_1}(U_1)+\textrm{Vol}^n_{g_2}(U_2)+\textrm{Vol}^{n-1}_{g_1}(\partial U_1)+\textrm{Vol}^{n-1}_{g_2}(\partial U_2)\right) \\&\quad +\textrm{Vol}^{n}_{g_1}(M_1\setminus U_1)+\textrm{Vol}^{n}_{g_2}(M_2\setminus U_2). \end{aligned}$$

## A Width Rigidity Theorem in All Dimensions á la Marques–Neves

In this section, we prove a version of Marques–Neves’ Theorem 2.3 for rotationally symmetric manifolds in *all dimensions* $n\geqslant 3$. We emphasize that Theorem 2.3 is manifestly a *three-dimensional* result, on account of the fact that it concerns the Simon–Smith width which has no known higher dimensional analog in generality (cf. Sect. 2.2). Rotational symmetry, however, allows us to introduce the related, weaker invariant $W^{\textrm{rot}}_g$ of Definition 2.5, which is clearly defined for every $n\geqslant 3$. With this quantity, we can state and prove a rotationally symmetric version of Theorem 2.3 which is valid in all dimensions $n\geqslant 3$; moreover, it requires *no* assumption on the Ricci curvature. The following is a restatement of Theorem B:

### Theorem 3.1

(Rigidity of Rotational Width in Rotational Symmetry) Let $n\geqslant 3$ and *g* be a rotationally symmetric Riemannian metric on the n-sphere, ${\mathbb {S}}^n$ such that

- $$\textrm{Scal}_g\geqslant n(n-1)$$;
- $$W^n_g \geqslant \omega _{n-1}$$,

where $W^3_g =W_g$ and $W^n_g=W^{\textrm{rot}}_g$ for $n\geqslant 4$. Then $(\mathbb {S}^n, g)$ is isometric to $(\mathbb {S}^n, g_{0}^n)$.

### Proof

Since $(\mathbb {S}^n,g)$ is rotationally symmetric, we can write it in the isometric form

$$\begin{aligned} \left( [0,D]\times \mathbb {S}^{n-1}, ds^2+f(s)^2g_0^{n-1}\right) \end{aligned}$$

where $f:[0,D]\rightarrow \mathbb {R}$ is a non-negative smooth function such that $f(0)=f(D)=0$ and $f'(0)=-f'(D)=1$. Now we apply Lemma 2.8. Therefore, we know that the canonical sweepout $\{\Sigma _{s}\}_{s\in [0,D]}$ contains exactly one minimal surface $\Sigma _{s_0}$ whose volume is $\omega _{n-1}f(s_0)$. By the definition of $W^n_g$ for each $n\geqslant 3$, we have that

$$\begin{aligned} \omega _{n-1}\leqslant W^n_g \leqslant \omega _{n-1}f(s_0)^{n-1}. \end{aligned}$$

We can now split the metric into two hemispheres. Without loss of generality, we will consider only one of the hemispheres. Consider the restricted metric $${\overline{g}}=ds^2+f(s)^2g_0^{n-1}$$ on the hemisphere $$\mathbb {S}^n_-=[0,s_0]\times \mathbb {S}^{n-1}$$, and note that $$\textrm{Scal}_{\overline{g}} \geqslant n(n-1)$$ and $$f'(s)\ne 0$$ on $$[0,s_0)$$ and $$f'(s_0)=0$$ (i.e., the boundary is a minimal surface).

Now we wish to change the coordinates. Let $r=f(s)$ and recall (2.1), i.e., the metric can now be expressed as

$$\begin{aligned} \bar{g}=\frac{dr^2}{V(r)} +r^2g_0^{n-1} \text { on } [0,R]\times {\mathbb {S}}^{n-1}. \end{aligned}$$

The goal now is to show that $V(r)=1-r^2$ and $R= 1$ which allows us to conclude that *g* is $g^n_0$, which we recall is the unit round metric on the *n*-sphere.

Since $f(s_0)\geqslant 1$, we see that $R\geqslant 1$. Recall the formula for scalar curvature (2.4). Using the scalar curvature bound, we see:

3.1 $$\begin{aligned} \textrm{Scal}_{\bar{g}} = \frac{n-1}{r}[(n-2)(1-V(r))-rV'(r)] \geqslant n(n-1). \end{aligned}$$

By multiplying (3.1) by the integrating factor $r^{n-2}$ and rearranging, we see that

3.2 $$\begin{aligned} (r^{n-2} (1-V(r)))'\geqslant nr^{n-1}. \end{aligned}$$

Next we integrate (3.2) from 0 to *R*, recalling from minimality that $V(R)=0$:

$$\begin{aligned} R^{n-2}\geqslant R^n \text { therefore } 1\geqslant R. \end{aligned}$$

We conclude then that $R=1$. Next we integrate (3.2) from 0 to *r* and rearrange to obtain that

$$\begin{aligned} 1-r^2 \geqslant V(r). \end{aligned}$$

If we instead integrate from *r* to $R\equiv 1$, it follows that

$$\begin{aligned} 1-r^{n-2}(1-V(r)) \geqslant 1-r^n \end{aligned}$$

and therefore

$$\begin{aligned} V(r) \geqslant 1-r^2. \end{aligned}$$

Thus, we conclude that $V(r)=1-r^2$ which finishes the proof. $\square$

## A Priori Diameter Estimate

In this section, we prove an explicit a priori upper bound for the diameter of a rotationally symmetric *n*-sphere with positive scalar curvature, non-negative Ricci curvature, and width bounded below. The general principle of interest in this argument appears in many different forms throughout geometric analysis, for example, in Bray’s Thesis [6] concerning the Penrose Inequality. Specifically, given a manifold $(M^n, g)$ with certain curvature bounds, one can seek special functions $f:M\rightarrow \mathbb {R}$ encoding geometric invariants of *M* which satisfy differential inequalities influenced by said curvature bounds. In [6], such functions of interest are the *isoperimetric profiles* of certain (*M*, *g*), which encode information about mass (Bray also used these techniques to give a new proof of Bishop’s Volume Theorem). In [14], Chodosh–Li–Minter–Stryker also used this general principle in the context of isoperimetry to establish volume bounds on three-dimensional $\mu$-bubbles with “spectral” curvature conditions, and in the context of Ricci lower bounds, this idea has long been applied by many authors with great success to, e.g., distance functions, Busemann functions, etc. Among many beautiful applications, we highlight here the seminal works of Colding and Cheeger–Colding in [11–13, 15].

Generally speaking, curvature conditions on the given (*M*, *g*) can impose convexity bounds on *f* via the differential inequalities, thereby controlling, e.g., the distance between roots of *f*. If *f* encodes geometric data of *M* in its roots, then we have found a way to bound certain invariants of *M*. In the situation at hand, our manifolds come with a canonical choice of function to consider—the warping function of the metric $f:[0,D]\rightarrow \mathbb {R}^{\geqslant 0}$ which, among other things, encodes the diameter in the size of its first positive root at *D*. As is well known, the condition $\textrm{Ric}_g\geqslant 0$ forces *f* to be *concave*, and together with $\textrm{Scal}_g\geqslant \Lambda >0$ and $W^n_g$ large enough, we can show that *f* is *quantitatively* concave, thereby controlling the size of *D*.

### Proposition 4.1

Let $n\geqslant 3$, $\Lambda >0$, $w_0>\sqrt{\frac{(n-1)(n-2)}{\Lambda }}{:=}1/\sqrt{\tilde{\Lambda }}$. Then there exists a $D_0=D_0(n, \Lambda , w_0)<\infty$ such that if

$$\begin{aligned}g=ds^2+f(s)^2g_0^{n-1} \text { with }s\in [0,D]\end{aligned}$$

is a smooth, rotationally symmetric metric on the *n*-hemisphere satisfying

- $$\textrm{Scal}_g\geqslant \Lambda >0$$;
- $$\textrm{Ric}_g\geqslant 0$$;
- $$f(0)\geqslant w_0>0$$, $$f'(0)=0$$, and $$f(D)=0$$,

then $D\leqslant D_0$. In particular, if $(\mathbb {S}^n, g)$ is rotationally symmetric and satisfies the above curvature conditions with $W^n_g\geqslant \omega _{n-1}w_0^{n-1}$, then $\textrm{diam}_g\leqslant 2D_0$.

Moreover, one may take the explicit value

$$\begin{aligned} D_0=\frac{4nw_0}{n-2}\cdot \frac{1}{\tilde{\Lambda }w_0^2-\log \left( {\tilde{\Lambda }} w_0^2\right) -1}. \end{aligned}$$

### Remark 4.2

The explicit value of $D_0$ is certainly not sharp. Nevertheless, the threshold value of $1/\sqrt{{\tilde{\Lambda }}}$ for the lower bound on $w_0$ is *sharp*, as can be seen by considering long and thin ellipsoids opening up to the constant scalar curvature $\Lambda$ cylinder. For clarity, consider a three-dimensional example “dual” to the example of Remark 2.6 (which has a clear extension to higher dimensions). Here, let

$$\begin{aligned} E_L:=\left\{ (x^2+y^2+z^2){\widetilde{\Lambda }}+w^2/L^2=1\right\} \end{aligned}$$

where we now consider the regime $L\gg 1$. For every such *L*, $E_L$ with its induced metric from Euclidean $\mathbb {R}^4$ has $\textrm{Scal}_g\geqslant \Lambda >0$, $\textrm{Ric}_g\geqslant 0$, and certainly no upper bound on diameter as we take $L\nearrow \infty$.

We also recall, in dimension $n=3$, that $8\pi /\Lambda$ is the upper threshold of area for a *stable*, embedded, closed, orientable minimal surface (necessarily a two-sphere) in an $(M^3, g)$ with $\textrm{Scal}_g\geqslant \Lambda >0$ (see, e.g., [36, Proposition A.1(i)]). This is exactly the area of the (unstable) 2-sphere realizing $W_g$ for $E_L$. If we let $\mathbb {S}^2\left( r\right)$ denote the constant curvature sphere of radius $r\leqslant 1/\sqrt{{\widetilde{\Lambda }}}$, and smoothly cap off the standard cylinders $\mathbb {S}^2\left( r\right) \times [-L, L]$, then any of the $S^2\left( r\right)$ cross sections in the core is a stable minimal surface of area $\leqslant 8\pi /\Lambda$, and again we have $\textrm{Scal}_g\geqslant \Lambda$, $\textrm{Ric}_g\geqslant 0$, and diameter unbounded above.

### Remark 4.3

It is easy to see that each of these curvature assumptions is necessary for a universal bound on diameter. Without a positive scalar curvature lower bound, the sole assumption of non-negative Ricci curvature cannot force the hemisphere to “close up” in a controlled manner, as one can see by considering capped-off cylinders of unbounded length. Without the assumption of non-negative Ricci curvature, strings of spheres joined by necks from the work of Gromov–Lawson [23] and Schoen–Yau [45] exist.

### Proof

Let $f:[0,D]\rightarrow \mathbb {R}^{\geqslant 0}$ be such a warping function for a metric *g* on the *n*-hemisphere. The curvature conditions in the hypotheses enforce the following differential inequalities. Recall (2.3) which tells us that the Ricci curvature bound implies.

4.1 $$\begin{aligned} f''(x)\leqslant 0 \end{aligned}$$

and recall (2.4) which tells us that the scalar curvature bound implies.

4.2 $$\begin{aligned} \Lambda \leqslant (n-1)(n-2)\left( \frac{1-f'(x)^2}{f(x)^2}-\frac{2}{(n-2)}\frac{f''(x)}{f(x)}\right) . \end{aligned}$$

Together with the assumption $f(0)\geqslant w_0$, (4.1) tells us that on [0, *D*]

$$\begin{aligned} f(x)\geqslant \left( 1-\frac{x}{D}\right) f(0)\geqslant \left( 1-\frac{x}{D}\right) w_0. \end{aligned}$$

Rearranging (4.2), plugging in the above lower bound on *f*(*x*), and using Lemma 2.1 tells us that on [0, *D*]

$$\begin{aligned} f''(x)\leqslant p(x){:=}\frac{n-2}{2}\left( \frac{1}{\left( 1-\frac{x}{D}\right) w_0}-{\tilde{\Lambda }} w_0\left( 1-\frac{x}{D}\right) \right) . \end{aligned}$$

Since $w_0>1/\sqrt{{\tilde{\Lambda }}}$, we have that $p(0)<0$ and $p'(x)>0$ so *p*(*x*) is increasing. Moreover,

$$\begin{aligned} \delta {:=}\sup \left\{ 0< x\leqslant D: p(x)<0 \text { on } [0, x)\right\} >0. \end{aligned}$$

Thus $p(\delta )=0$ which explicitly gives

$$\begin{aligned} \delta =D\left( 1-\frac{1}{w_0\sqrt{{\tilde{\Lambda }}}}\right) . \end{aligned}$$

Now, recalling that $f'(0)=0$, $f'(D)=-1$, and $f''(x)\leqslant 0$, we compute from the above that

$$\begin{aligned} -1=\int _0^D f''(s)ds\leqslant \int _0^\delta p(s)ds=\frac{n-2}{4nw_0}\left( 1-{\tilde{\Lambda }} w_0^2+\log \left( {\tilde{\Lambda }} w_0^2\right) \right) D<0 \end{aligned}$$

from which the claim follows. $\square$

Using the quantitative control $f''(x)\leqslant p(x)<0$ on $[0,\delta )$ from the proof of Proposition 4.1, we can immediately prove the following version of Lemma 2.8 from Sect. 2, which replaces control on $\textrm{MinA}_g$ with control on $W^n_g$. We will use this in the proof of Theorem C.

### Lemma 4.4

Let $n\geqslant 3$. Then there exists an $c(n)>0$ such that for any $0<\varepsilon <c(n)$ and

$$\begin{aligned}g=ds^2+f(s)^2g_0^{n-1} \text { with }s\in [0,D]\end{aligned}$$

is smooth, rotationally symmetric metric on the *n*-sphere $\mathbb {S}^n$ satisfying

- $$\textrm{Scal}_g\geqslant n(n-1)(1-\varepsilon )^2$$;
- $$\textrm{Ric}_g\geqslant 0$$;
- $$W^n_g\geqslant \omega _{n-1}(1-\varepsilon )^{n-1}$$,

then the canonical sweepout $\{\Sigma _s\}_{s\in [0, D]}$ of $(\mathbb {S}^n,g)$ contains exactly one minimal hypersurface, which is necessarily unstable.

### Proof

As in the proof of Lemma 2.8, since $f(0)=f(D)=0$, there is some minimal sphere $\Sigma _{s_0}$ where $f(s_0)=\max _{s\in [0,D]} f(s)\geqslant (1-\varepsilon )$ and $f'(s_0)=0$, where the lower bound on $f(s_0)$ follows from the condition

$$\begin{aligned}\omega _{n-1}f(s_0)^{n-1}=\textrm{Vol}^{n-1}_g(\Sigma _{s_0})\geqslant W_g^n\geqslant \omega _{n-1}(1-\varepsilon )^{n-1}.\end{aligned}$$

Since $\textrm{Ric}_g\geqslant 0$, we have that $f''(x)\leqslant 0$ on [0, *D*], and recalling the proof of Proposition 4.1 it follows (modulo a coordinate shift $0\mapsto s_0$) that $f''(s_0)\leqslant p(s_0)<0$ provided $\varepsilon >0$ is small enough to begin with. Thus, $f'(s)<0$ for every $s\in (s_0, D]$, and there are no further minimal spheres in $\{\Sigma _s\}_{s\in (s_0, D]}$. A similar analysis shows that there are none in $\{\Sigma _s\}_{s\in [0, s_0)}$, so we are done. $\square$

## Stability of the Width: Proofs of Theorem A and Theorem D

In this section, we prove Theorem A and Theorem D, which will follow immediately from the following theorems, Lemma 2.8, Proposition 4.1 and Lemma 4.4. Therefore, we will be considering rotationally symmetric metrics on the *n*-sphere $\mathbb {S}^n$, $n\geqslant 3$, satisfying:

5.1 $$\begin{aligned}&\hspace{.25in}\bullet \,\textrm{diam}_g(\mathbb {S}^n)\leqslant D;&\end{aligned}$$

5.2 $$\begin{aligned}&\hspace{.25in}\bullet \,\textrm{Scal}_g\geqslant n(n-1)(1-\varepsilon )^2,&\end{aligned}$$

5.3 $$\begin{aligned}&\hspace{.25in}\bullet \,\textrm{MinA}_g\geqslant \omega _{n-1}(1-\varepsilon )^{n-1}.&\end{aligned}$$

### Theorem 5.1

Fix $n\geqslant 3$, $D>0$, and $\delta >0$. There exists an $\varepsilon =\varepsilon (n, \delta , D)>0$ such that if *g* is a rotationally symmetric metric on the *n*-sphere $\mathbb {S}^n$ satisfying (5.1), (5.2), and (5.3) then $\textrm{d}_{\mathcal{V}\mathcal{F}}((\mathbb {S}^n, g), (\mathbb {S}^n, g_{0}^n))\leqslant \delta$.

Without any further assumptions, and notably without any further curvature bounds, we obtain the following more-or-less equivalent rephrasing of Theorem 5.1 in terms of the Gromov–Hausdorff distance. In this case, the possible formation of spines at the poles of the spheres is not controlled by the Gromov–Hausdorff topology, so we must excise this possibly “bad” set to get Gromov–Hausdorff convergence (this technique has been used to great effect in stability problems before–see eg. Dong–Song [20], Hirsch–Zhang [26], and Bryden–Chen [9]). The following is a restatement of Theorem D:

### Theorem 5.2

Fix $n\geqslant 3$, $D>0$, and $\delta >0$. There exists an $\varepsilon =\varepsilon (n, \delta , D)>0$ such that if *g* is a rotationally symmetric metric on the *n*-sphere $\mathbb {S}^n$ satisfying (5.1), (5.2), and (5.3) then there exists a smooth domain $Z\subset \mathbb {S}^n$ with at most two connected components satisfying

$$\begin{aligned}\textrm{Vol}_g^n(Z)+\textrm{Vol}_g^{n-1}(\partial Z)\leqslant \delta ,\end{aligned}$$

so that $$\textrm{d}_{\textrm{GH}}((\mathbb {S}^n\setminus Z, g), (\mathbb {S}^n, g_{0}^n))\leqslant \delta $$.

First, we prove a lemma.

### Lemma 5.3

Let $\varepsilon \in (0,1)$ and $g_\varepsilon$ be a Riemannian metric on the *n*-hemisphere such that the following hold:

5.4 $$\begin{aligned}&\hspace{.25in} \bullet \, g_\varepsilon \text { is rotationally symmetric, i.e, } g_\varepsilon =ds^2+f_\varepsilon (s)^2g_0^{n-1} \text { on } [0,\tilde{S}_\varepsilon ]\times \mathbb {S}^{n-1};&\end{aligned}$$

5.5 $$\begin{aligned}&\hspace{.25in}\bullet \, f_\varepsilon '(s)>0 \text { on } [0,\tilde{S}_\varepsilon ) \text { and } f'_\varepsilon (\tilde{S}_\varepsilon )=0 \text {, i.e., the boundary is a minimal surface}.&\end{aligned}$$

Then we may use $r:=f_\varepsilon (s)$ itself as the coordinate, and write

$$\begin{aligned} g_\varepsilon =\frac{dr^2}{V_\varepsilon (r)}+r^2g_0^{n-1} \text { on } [0,R_\varepsilon ]\times \mathbb {S}^{n-1}. \end{aligned}$$

Furthermore, if (5.2) and (5.3) are satisfied then

$$\begin{aligned} 1-\varepsilon \leqslant R_\varepsilon \le \frac{1}{1-\varepsilon } \text { and } 1-\varepsilon \leqslant \tilde{S}_\varepsilon . \end{aligned}$$

### Proof

By (5.2) and (2.4),

$$\begin{aligned} \textrm{Scal}_{g_\varepsilon }=\frac{n-1}{r^2}\left( (n-2)(1 - V_{\varepsilon }(r)) - r V_{\varepsilon }'(r)\right) \geqslant n(n-1)(1-\varepsilon )^2 \end{aligned}$$

which after suitable rearrangement becomes

$$\begin{aligned} \left( r^{n-2}(1-V_{\varepsilon }(r))\right) '\geqslant nr^{n-1}(1-\varepsilon )^2. \end{aligned}$$

We note that (5.5) we have that $V_{\varepsilon }(R_{\varepsilon })=0$. If we integrate both sides from 0 to $R_{\varepsilon }$ using $V_{\varepsilon }(R_{\varepsilon }) = 0$, then we see $R_{\varepsilon }\le \frac{1}{1-\varepsilon }$. The lower bound on $R_{\varepsilon }$ follows from $\textrm{MinA}_{g_k}$ lower bound and the fact the boundary is a minimal surface with area $\omega _{n-1}R_\varepsilon ^{n-1}$. Finally, by pairing the $MinA_{g_{\varepsilon }}$ lower bound with Lemma 2.1, we also obtain the lower bound on $\tilde{S}_\varepsilon$. $\square$

We will prove these theorems by way of contradiction. That is we will consider a sequence of smooth metric tensors on $\mathbb {S}^n$,

$$\begin{aligned} g_k=ds^2+f_k(s)^2g_0^{n-1}\text { on } [0,S_k]\times \mathbb {S}^{n-1} \text { for } k=1, 2, \ldots \end{aligned}$$

satisfying:

5.6 $$\begin{aligned}&\hspace{.25in}\bullet \,\textrm{diam}_{g_k}(\mathbb {S}^n)\leqslant D;&\end{aligned}$$

5.7 $$\begin{aligned}&\hspace{.25in}\bullet \,\textrm{Scal}_{g_k}\geqslant n(n-1)(1-k^{-1})^2,&\end{aligned}$$

5.8 $$\begin{aligned}&\hspace{.25in}\bullet \,\textrm{MinA}_{g_k}\geqslant \omega _{n-1}(1-k^{-1})^{n-1}.&\end{aligned}$$

such that the conclusion of each theorem is false respectively.

In the following discussion, we will obtain general estimates on *any* sequence of smooth, rotationally symmetric metrics

5.9 $$\begin{aligned} g_k=ds^2+f_k(s)^2g_0^{n-1}\text { on } [0,S_k]\times \mathbb {S}^{n-1} \text { for } k=1, 2, \ldots \end{aligned}$$

which depend *only* on properties (5.6), (5.7), and (5.8).

**Fig. 1:**
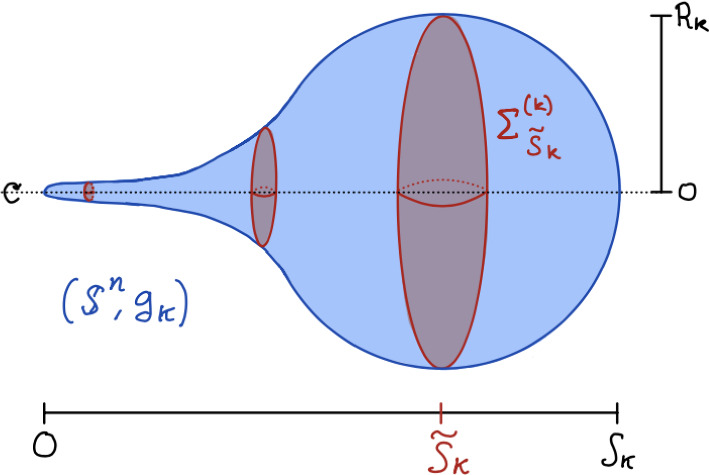
$(\mathbb {S}^n, g_k)$ with the minimal sphere $\Sigma ^{(k)}_{\tilde{S}_k}$

Recall by Lemma 2.8 (and Remark 2.9), for all $k> \frac{1}{c(n)}$, there is only one minimal hypersurface $\Sigma ^{(k)}_{\tilde{S}_k}=\{\tilde{S}_k\}\times \mathbb {S}^{n-1}$ in the canonical sweepout of $(\mathbb {S}^n, g_k)$, which divides $(\mathbb {S}^n, g_k)$ into two connected hemispheres which we denote by $(\mathbb {S}^n_\pm , g_k)$, which satisfy (5.4) and (5.5) (see Fig. 1). To simplify exposition, in the following sequence of lemmas we will only explicitly work on $(\mathbb {S}^n_-, g_k)$ since the situation with $(\mathbb {S}^n_+, g_k)$ is handled nearly identically.

Since $g_k$ satisfy (5.4) and (5.5), we can introduce $(r,\theta )$ coordinates. Recall (2.1) and so on the hemisphere $(\mathbb {S}^n_-, g_k)$ we can express the metric as

5.10 $$\begin{aligned} \left( \mathbb {S}^n_-, g_k\right)&=_{isom}\left( \left[ 0,\tilde{S}_k\right) \times \mathbb {S}^{n-1}, \quad g_k=ds^2+f_k(s)^2g_0^{n-1}\right) \end{aligned}$$

5.11 $$\begin{aligned}&=_{isom}\left( \left[ 0,R_k\right) \times \mathbb {S}^{n-1}, \quad g_k=\frac{1}{V_{k,-}(r)}dr^2+r^2g_0^{n-1}\right) . \end{aligned}$$

We also recall that this means *r* is related to *s* via the formula $$r=f_k(s)$$, and that on $$[0, R_k)$$

$$\begin{aligned} V_{k,-}(r)=\left( f_k'\left( \left( f_k\vert _{\left[ 0,\tilde{S}_k\right) }\right) ^{-1}(r)\right) \right) ^2. \end{aligned}$$

Now by Lemma 5.3 and the fact $S_k\leqslant {\textrm{diam}}_g({\mathbb {S}}^n)\leqslant D$, we have that

5.12 $$\begin{aligned} 1-k^{-1} \leqslant R_k \leqslant \frac{1}{1-k^{-1}} \text { and } 1-k^{-1} \leqslant \tilde{S}_k \le S_k \le D. \end{aligned}$$

For every $k\geqslant \frac{1}{c(n)}$, we extend (if necessary) $V_{k,-}(r)$ from $[0, R_k]$ to [0, 1] constantly by 0, and we extend $f_k(s)$ from $[0, \tilde{S}_k]$ to [0, *D*] constantly by $R_k$. These extensions are smooth everywhere on their now fixed domains of definition, except at the points $R_k$ and $\tilde{S}_k$, respectively, where they are continuous. By (5.12), we can assume that up to a subsequence, $\tilde{S}_k\nearrow \tilde{S}\in [1,D]$.

Using the scalar curvature (5.7) and Lemma 5.3, we may now prove our two most fundamental estimates on our metric tensors, which give pointwise convergence to the round sphere. The first estimate will eventually allow us to prove volume convergence (Lemma 5.10) and together with the second estimate we will be able to establish all of the other estimates needed (Lemmas 5.7, 5.8, 5.9) to apply Lakzian–Sormani’s Theorem 2.12 to obtain the desired contradiction.

### Lemma 5.4

(Fundamental Metric Estimates (I)) Assume (5.7). Fix $0<\eta <1$. For every $k\geqslant 1$ large enough, we have the following uniform estimate for $r\in [\eta ,1]$:

$$\begin{aligned} \left| V_{k,-}(r)-V_{0}(r)\right| \leqslant \Psi (k^{-1}:\eta ) \end{aligned}$$

where we recall that $V_{0}(r)=1-r^2$. Therefore if $0<\rho<<1$, we have that

$$\begin{aligned} \left| \frac{V_{k,-}(r)}{V_{0}(r)}-1\right| \leqslant \Psi (k^{-1}:\eta , \rho )\quad \text {on}\quad [\eta , 1-\rho ]. \end{aligned}$$

### Proof

By (2.4) and the scalar curvature lower bounds (5.7) we have the following ordinary differential inequality for $V_{k,-}$ on $[0,R_k]$:

$$\begin{aligned} n(n-1)(1-k^{-1})^2\leqslant \textrm{Scal}_{g_k}=\frac{n-1}{r^2}\left( (n-2)(1-V_{k,-}(r))-rV_{k,-}'(r)\right) , \end{aligned}$$

or in other words

$$\begin{aligned} V_{k,-}'(r)+\frac{n-2}{r}V_{k,-}(r)\leqslant \frac{n-2}{r}- n(1-k^{-1})^2r. \end{aligned}$$

After multiplying this last line by the integrating factor $r^{n-2}$ and integrating from 0 to $r\in (0,R_k]$, we obtain the upper bound

$$\begin{aligned} V_{k,-}(r)\leqslant 1-(1-k^{-1})^2r^2\quad \text {on } [0,R_k]. \end{aligned}$$

If we instead integrate from $r\in [0,R_k)$ to $R_k$ and recall that $V_{k,-}(R_k)=0$ by minimality, we obtain the lower bound

$$\begin{aligned} (1-(1-k^{-1})^2r^2)+\frac{(1-k^{-1})^2R_k^n-R_k^{n-2}}{r^{n-2}}\leqslant V_{k,-}(r)\quad \text {on } (0,R_k]. \end{aligned}$$

Notice that by (5.12) the second term on the left hand side could degenerate to $-\infty$ as $r\searrow 0$, but that on $[\eta , R_k]$ it is always bounded and decays to 0 uniformly as $k\rightarrow \infty$. Therefore, recalling the definition of $V_{0}(r)=1-r^2$ on [0, 1], we can easily wrap these estimates into the form

$$\begin{aligned}\left| V_{k,-}(r)-V_{0}(r)\right| \leqslant \Psi (k^{-1}:\eta )\quad \text {on } [\eta , 1].\end{aligned}$$

The last estimate in the lemma follows from the first, if we pair it with the bounds $1\geqslant V_{0}(r)\geqslant 1-(1-\rho )^2$ on $[\eta , 1-\rho ]$. $\square$

### Lemma 5.5

(Fundamental Metric Estimates (II)) Assume (5.6) and (5.7). For every $k\geqslant 1$ large enough, we have the following uniform estimate for $s\in [\tilde{S}-1,\tilde{S}]$ (recall that $\tilde{S}=\lim _{k\rightarrow \infty }\tilde{S}_k$):

$$\begin{aligned} \left| \frac{f_k(s)}{f_{0}\left( s+\left( \frac{\pi }{2}-\tilde{S}\right) \right) }-1\right| \leqslant \Psi (k^{-1}) \end{aligned}$$

where we recall that $f_{0}(s)=\sin (s)$. Therefore, for all large *k*, $f_k(\tilde{S}_k-1)\leqslant \frac{3}{4}$ so that the two coordinate systems

$$\begin{aligned} (r,\theta )\in U_{k,\eta , -}:=\left( \eta , R_k-\frac{1}{100}\right) \times \mathbb {S}^{n-1}\quad \text {and}\quad (s,\theta )\in E_{k,-}:=\left( \tilde{S}_k-\frac{99}{100}, \tilde{S}_k\right] \times \mathbb {S}^{n-1} \end{aligned}$$

cover all of $(\mathbb {S}^n_-, g_k)$ except the “small” region $[0,\eta ]\times \mathbb {S}^{n-1}$ in the $(r,\theta )$ coordinates, and where both of these coordinate charts enjoy the estimates of this and the previous lemma.

### Proof

We first observe that by (5.6) (the upper diameter bound *D*) and the a priori Lipschitz bound of Lemma 2.1, the Arzelá–Ascoli Theorem guarantees that a subsequence of the $f_k$ converges uniformly on $[0,\tilde{S}]$ to a non-negative 1-Lipschitz function $f_\infty$ which clearly satisfies $f_\infty (0)=0$ and $f_\infty (\tilde{S})=1$ (by (5.12)). In fact, we claim that $f_\infty$ satisfies the following partial boundary value problem, from which the result follows easily:

$$\begin{aligned}{\left\{ \begin{array}{ll} f_\infty '(s)^2+f_\infty (s)^2=1 &  \text {on}\quad (\tilde{S}-1,\tilde{S})\\ f_\infty (\tilde{S})=1. &  \end{array}\right. } \end{aligned}$$

To establish this, recall that $V_{k,-}(f_k(s))=(f_k'(s))^2$. Fix an arbitrary $0<\eta <1$. For any $s\in (\tilde{S}-\eta , \tilde{S})$, for all large enough *k* we also have $s\in (\tilde{S}_k-\eta , \tilde{S}_k)$ where $f_k$ is smooth. By Lemma 2.1, we can estimate that

$$\begin{aligned} f_k(s)=R_k-\int _{s}^{\tilde{S}_k} f'_k(\xi )d\xi \geqslant (1-k^{-1})-(\tilde{S}_k-s)\geqslant 1-\eta -k^{-1}\geqslant \frac{1-\eta }{2}>0 \end{aligned}$$

for all large enough *k* (depending on $\eta$). By the uniform convergence of the $V_{k,-}$ on $[(1-\eta )/2, 1]$ just proven in Lemma 5.4, we therefore see that we also have uniform convergence of the $f_k'(s)=\left( V_{k,-}(f_k(s))\right) ^{1/2}$ on $[\tilde{S}-\eta , \tilde{S}-\rho ]$ for any $0<\rho<<1$. Thus,

$$\begin{aligned} |f_k'(s)^2-V_{k,-}(f_k(s))|\leqslant \Psi (k^{-1}: \eta , \rho )\quad \text {on}\quad [\tilde{S}-\eta , \tilde{S}-\rho ] \end{aligned}$$

and so by Lemma 5.4 and the fact that $f_k\rightarrow f_\infty$ uniformly on $[0,\tilde{S}]$,

$$\begin{aligned} |f_k'(s)^2+f_\infty (s)^2-1|\leqslant \Psi (k^{-1}: \eta , \rho )\quad \text {on}\quad [\tilde{S}-\eta , \tilde{S}-\rho ]. \end{aligned}$$

Thus, by sending $k\rightarrow \infty$ we obtain that $f_\infty$ is differentiable and

$$\begin{aligned} f_\infty '(s)^2+f_\infty (s)^2=1\quad \text {on}\quad [\tilde{S}-\eta , \tilde{S}-\rho ], \end{aligned}$$

from which smoothness follows. Sending $\eta \nearrow 1$ and $\rho \searrow 0$ establishes the claim, from which we see that

$$\begin{aligned} f_\infty (s)=\sin \left( s+\left( \frac{\pi }{2}-\tilde{S}\right) \right) \quad \text {on}\quad [\tilde{S}-1,\tilde{S}]. \end{aligned}$$

The conclusion of the lemma now follows readily. $\square$

### Remark 5.6

**Fig. 2:**
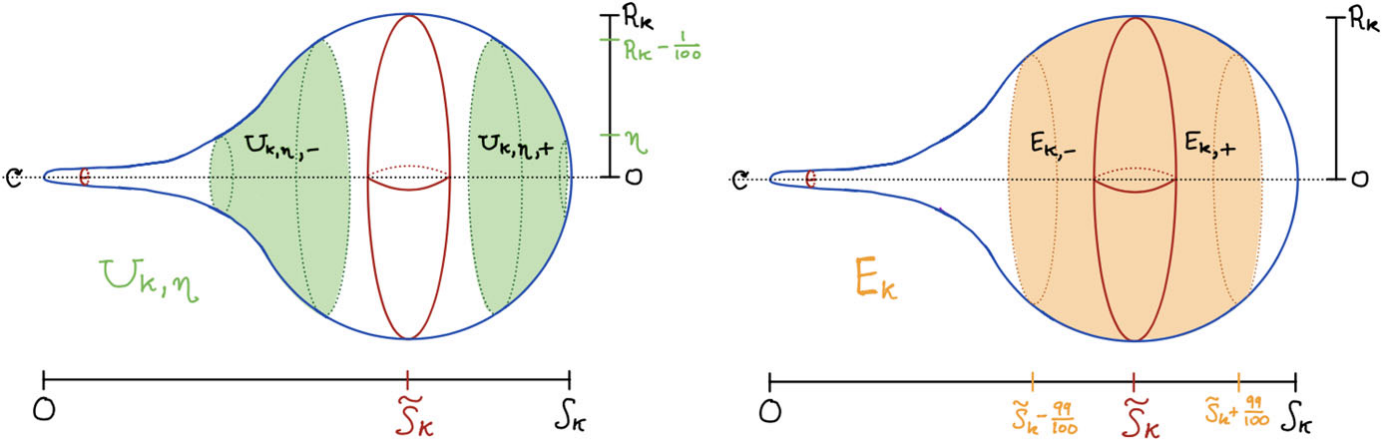
The charts $U_{k,\eta }$ and ${E_k}$

Our arguments require us to use both of these coordinate systems in order to cover enough of the manifold to achieve global convergence. Notice that Lemma 5.5 addresses a region of definite size around the largest sphere in the canonical sweepout of each $(\mathbb {S}^n, g_k)$, and tells us that inside this region we asymptotically see the geometry of the round sphere. However, the possibility of spine formation away from the minimal sphere causes the estimates in these coordinate charts to break down. Nevertheless, Lemma 5.4 and the uniform diameter bound give us enough control on the rest of the manifold to make up for this. See Fig. 2.

In the following sequence of lemmas, we estimate the various quantities appearing in the Lakzian–Sormani estimates of Theorem 2.12 using Lemmas 5.4 and 5.5. We also return to global setting on all of $\mathbb {S}^n$, having similarly carried out the analogous estimates on the hemispheres $(\mathbb {S}_+^n, g_k)$, which we now express in the following useful coordinate parametrizations:

$$\begin{aligned} \left( \mathbb {S}^n_+, g_k\right)&=_{isom}\left( \left( \tilde{S}_k, S_k\right] \times \mathbb {S}^{n-1}, \quad g_k=ds^2+f_k(s)^2g_0^{n-1}\right) \\  &=_{isom}\left( \left( 2-R_k,2\right] \times \mathbb {S}^{n-1}, \quad g_k=\frac{1}{V_{k,+}(2-r)}dr^2+(2-r)^2g_0^{n-1}\right) \end{aligned}$$

where $2-r=f_k(S_k-s)$. Using the notation established in Lemma 5.5, we thus work in the following charts for $(\mathbb {S}^n, g_k)$ (see again Fig. 2):

$$\begin{aligned}&(r,\theta )\in U_{k,\eta }:=U_{k,\eta ,-}\sqcup U_{k,\eta , +}=\left( \left( \eta , R_k-\frac{1}{100}\right) \cup \left( 2-R_k+\frac{1}{100}, 2-\eta \right) \right) \times \mathbb {S}^{n-1}\\&(s,\theta )\in E_k:=E_{k,-}\cup E_{k,+}=\left( \tilde{S}_k-\frac{99}{100}, \tilde{S}_k+\frac{99}{100}\right) \times \mathbb {S}^{n-1}, \end{aligned}$$

where the restriction of $g_k$ (defined in $(r,\theta )$ coordinates for $r\in [0, 2R_k]\setminus \{R_k\}$) has the following coordinate expressions:

$$\begin{aligned} g_k\vert _{p}={\left\{ \begin{array}{ll} \frac{1}{V_{k,-}(r)}dr^2+r^2g_0^{n-1} &  \text {if } p=(r,\theta )\in U_{k, \eta , -}\subset U_{k,\eta }\\ ds^2+f_k(s)^2g_0^{n-1} &  \text {if } p=(s,\theta )\in E_k \\ \frac{1}{V_{k,+}(2-r)}dr^2+(2-r)^2g_0^{n-1} &  \text {if } p=(r,\theta )\in U_{k, \eta , +}\subset U_{k,\eta }.\\ \end{array}\right. } \end{aligned}$$

We also define the fixed charts

$$\begin{aligned}&(r,\theta )\in U_\eta :=U_{\eta ,-}\sqcup U_{\eta , +}=\left( \left( \eta , \frac{9}{10}\right) \cup \left( \frac{11}{10}, 2-\eta \right) \right) \times \mathbb {S}^{n-1}\\&(s,\theta )\in E:=E_-\cup E_+=\left( \tilde{S}-\frac{9}{10}, \tilde{S}+\frac{9}{10}\right) \times \mathbb {S}^{n-1}, \end{aligned}$$

where the restriction of $g_{0}^n$ (defined in $(r,\theta )$ coordinates for $r\in [0,2]\setminus \{1\}$) has the following coordinate expressions:

$$\begin{aligned} g_{0}^n\vert _{p}= {\left\{ \begin{array}{ll} \frac{1}{1-r^2}dr^2+r^2g_0^{n-1} &  \text {if } p=(r,\theta )\in U_{\eta , -}\subset U_{\eta }\\ ds^2+\sin \left( s+\left( \frac{\pi }{2}-\tilde{S}\right) \right) ^2g_0^{n-1} &  \text {if } p=(s,\theta )\in E \\ \frac{1}{1-(2-r)^2}dr^2+(2-r)^2g_0^{n-1} &  \text {if } p=(r,\theta )\in U_{\eta , +}\subset U_{\eta }.\\ \end{array}\right. } \end{aligned}$$

Finally, we let

$$\begin{aligned}\Omega _\eta {:=}(\eta , 2-\eta )\times \mathbb {S}^{n-1} \end{aligned}$$

denote a spherical band. For all $k\geqslant \frac{1}{c(n)}$ so large that $|R_k-1|, |\tilde{S}_k-\tilde{S}|<\min \{\frac{1}{1000},\frac{\eta }{2}\}$, Lemma 5.5 guarantees that the charts $(U_{k,\eta }, g_k)$ and $(E_k, g_k)$ together cover $\Omega _\eta$ with the pullback of $g_k$ by the natural inclusion induced by the $(r,\theta )$ coordinates, which we write as

$$\begin{aligned} \Omega _{k,\eta }{:=}(\Omega _\eta , g_k) \hookrightarrow (\mathbb {S}^n, g_k). \end{aligned}$$

Corresponding to this is, of course, the following subregion of the round sphere:

$$\begin{aligned} \Omega _{{g_0^n}, \eta }:=(\Omega _\eta , g_{0}^n)\hookrightarrow (\mathbb {S}^n, g_{0}^n). \end{aligned}$$

Lemmas 5.4 and 5.5 then allow us to uniformly compare the components of $g_k$ and $g_{0}^n$ on $\Omega _\eta$ to easily obtain the first estimate needed for Lakzian–Sormani’s Theorem 2.12:

### Lemma 5.7

($C^0$ Cheeger–Gromov Convergence) Assume (5.6) and (5.7). For all $k\geqslant \frac{1}{c(n)}$ large enough,

$$\begin{aligned} 1-\Psi (k^{-1}:\eta )\leqslant \frac{g_k(v,v)}{g_{0}^n(v,v)}\leqslant 1+\Psi (k^{-1}:\eta ) \end{aligned}$$

for every $v\in T\Omega _\eta$ (where we have omitted the pullback maps to $\Omega _\eta$ from the notation for readability).

### Proof

Fix $v_p\in T_p\Omega _\eta$. If $p=(r,\theta )\in U_\eta$, then for all large enough $k\geqslant \frac{1}{c(n)}$ and Lemma 5.4

$$\begin{aligned} \frac{g_k(v,v)}{g_{0}^n(v,v)}&=\frac{\frac{1}{V_k(r)}dr^2(v,v)+r^2g_0^{n-1}(v,v)}{\frac{1}{\sqrt{1-r^2}}dr^2(v,v)+r^2g_0^{n-1}(v,v)}\\&\leqslant \frac{(1+\Psi (k^{-1}:\eta ))\left( \frac{1}{\sqrt{1-r^2}}dr^2(v,v)+r^2g_0^{n-1}(v,v)\right) }{\frac{1}{\sqrt{1-r^2}}dr^2(v,v)+r^2g_0^{n-1}(v,v)}\\&=1+\Psi (k^{-1}:\eta ) \end{aligned}$$

and similarly for the other inequality. Likewise, if it happens that $p=(s,\theta )\in E$, then for every $k\geqslant \frac{1}{c(n)}$ large enough and Lemma 5.5

$$\begin{aligned} \frac{g_k(v,v)}{g_{0}^n(v,v)}&=\frac{ds^2(v,v)+f_k(s)^2g_0^{n-1}(v,v)}{ds^2(v,v)+\sin \left( s+\left( \frac{\pi }{2}-\tilde{S}\right) \right) ^2g_0^{n-1}(v,v)}\\&\leqslant \frac{(1+\Psi (k^{-1}:\eta ))\left( ds^2(v,v)+\sin \left( s+\left( \frac{\pi }{2}-\tilde{S}\right) \right) ^2g_0^{n-1}(v,v)\right) }{ds^2(v,v)+\sin \left( s+\left( \frac{\pi }{2}-\tilde{S}\right) \right) ^2g_0^{n-1}(v,v)}\\&=1+\Psi (k^{-1}:\eta ) \end{aligned}$$

and similarly for the other inequality, as desired. $\square$

For the next estimate, given $\Omega \subset (M,g)$ we recall the quantity

$$\begin{aligned} D_{\Omega }=\sup \{\textrm{diam}_{M}(W): \hbox { W is a connected component of}\ \Omega \}. \end{aligned}$$

Since $S_k=\textrm{diam}_{g_k}(\mathbb {S}^n)\leqslant D$, we immediately obtain the following lemma:

### Lemma 5.8

(Estimating $D_{\Omega _{k, \eta }}$, $D_{\Omega _{{g_0^n},\eta }}$, and *a*) Assume (5.6).For all $k\geqslant \frac{1}{c(n)}$ large enough,

$$\begin{aligned} D_{\Omega _{k, \eta }}\leqslant D \qquad \text {and}\qquad D_{\Omega _{{g_0^n},\eta }}\leqslant \pi . \end{aligned}$$

Therefore, the parameter *a* in the statement of Theorem 2.12 may be taken such that $a\leqslant \Psi (k^{-1}).$

It may be worth noting that one can easily obtain sharper estimates for $D_{\Omega _{k,\eta }}$ and thus *a* in the above by explicitly constructing curves in $(\mathbb {S}^n,g_k)$ between pairs of points $x,y\in \Omega _{k,\eta }$ and bounding their lengths using Lemmas 5.4 and 5.5, instead of cheaply using the uniform diameter bound. In this case, we would be able to ensure a choice of *a* such that that $a\leqslant \Psi (k^{-1}, \eta )$ (recall from Subsect. 1.1 that this means $\Psi \rightarrow 0$ as $k\rightarrow \infty$ *and* $\eta \searrow 0$). We carry such an argument out in the following:

### Lemma 5.9

(Estimating $\lambda$, *h*, and ${\overline{h}}$) Assume (5.6) and (5.7). For all large enough $k\geqslant \frac{1}{c(n)}$,

$$\begin{aligned} \lambda _k:=\sup _{x,y\in \Omega _\eta } \left| d_{g_k}(x,y)-d_{g_{0}^n}(x,y)\right| \leqslant \Psi (k^{-1}, \eta ). \end{aligned}$$

Therefore, we also have $0\leqslant h\leqslant \Psi (k^{-1}, \eta )$ and $0\leqslant {\overline{h}}\leqslant \Psi (k^{-1}, \eta )$.

### Proof

Fix any $x,y\in \Omega _\eta$. We first prove that

$$\begin{aligned} d_{g_k}(x,y)-d_{g_{0}^n}(x,y)\leqslant \Psi (k^{-1}, \eta ). \end{aligned}$$

To do so, let $\gamma$ be a minimizing $g_{0}^n$ geodesic in $\mathbb {S}^n$ connecting *x* to *y*, which may certainly leave $\Omega _{{g_0^n},\eta }$. Let ${\tilde{\gamma }}$ be the piecewise smooth curve from *x* to *y* contained in the closure of $\Omega _{{g_0^n},\eta }$ obtained by replacing the single connected portion of $\gamma$ outside $\Omega _{{g_0^n},\eta }$ with an intrinsically minimizing great circle arc in $\partial \Omega _{{g_0^n},\eta }$. This yields a piecewise smooth curve in $\Omega _\eta$ and thus in $\Omega _{k,\eta }$ which we continue to denote as ${\tilde{\gamma }}$. By the $C^0$ Cheeger–Gromov convergence of Lemma 5.7 (applied on, say, $\Omega _{\eta /2}$), we have that

$$\begin{aligned} d_{g_k}(x,y)&\leqslant L_{g_k}({\tilde{\gamma }})\leqslant L_{g_{0}^n}({\tilde{\gamma }})+\Psi (k^{-1})\leqslant L_{g_{0}^n}(\gamma )+\Psi (\eta )+\Psi (k^{-1})\\&=d_{g_{0}^n}(x,y)+\Psi (k^{-1}, \eta ). \end{aligned}$$

Next we prove the opposite inequality

$$\begin{aligned} d_{g_{0}^n}(x,y)-d_{g_k}(x,y)\leqslant \Psi (k^{-1}, \eta ) \end{aligned}$$

by fixing a $g_k$ geodesic $\gamma : [0,1]\rightarrow \mathbb {S}^n$ from *x* to *y*. This curve $\gamma$ may just as well leave $\Omega _{k, \eta }$, but since it must begin and end in $\Omega _{k,\eta }$ there is a maximal set of times of the particular form $\mathcal {I}:=[0,t_1)\cup (t_2, 1]\subset [0,1]$ so that $\gamma ':=\gamma \vert _\mathcal {I}\subset \Omega _{k,\eta }$ We simply replace the entire portion of $\gamma$ between $\gamma (t_1)$ and $\gamma (t_2)$ with an intrinsically minimizing great circle arc in $\partial \Omega _{{g_0^n},\eta }$ to similarly obtain a new curve ${\tilde{\gamma }}$ lying in the closure of $\Omega _{k,\eta }$. We therefore estimate as above that for all large $k\geqslant \frac{1}{c(n)}$,

$$\begin{aligned} d_{g_k}(x,y)&=L_{g_k}(\gamma )\geqslant L_{g_k}(\gamma ')\geqslant L_{g_{0}^n}(\gamma ')-\Psi (k^{-1})\\  &\geqslant L_{g_{0}^n}({\tilde{\gamma }})-\Psi (\eta )-\Psi (k^{-1})\geqslant d_{g_{0}^n}(x,y)-\Psi (k^{-1}, \eta ), \end{aligned}$$

where we have used the fact that the added portion in $\partial \Omega _\eta$ has round length less than $\pi \eta$. $\square$

Moving on, we establish convergence of the various volume quantities appearing in the estimates of Theorem 2.12:

### Lemma 5.10

(Volume Convergence) Assume (5.6) and (5.7). For all $k\geqslant \frac{1}{c(n)}$ large enough, we have that

5.13 $$\begin{aligned}&\hspace{.25in}\bullet \,|\textrm{Vol}_{g_k}^n(\Omega _{k,\eta })-\textrm{Vol}_{g_{0}^n}^n(\Omega _{{g_0^n},\eta })|\leqslant \Psi (k^{-1}).&\end{aligned}$$

5.14 $$\begin{aligned}&\hspace{.25in}\bullet \,\textrm{Vol}^n_{g_k}(\mathbb {S}^n\setminus \Omega _{k,\eta })\leqslant \Psi (\eta ).&\end{aligned}$$

5.15 $$\begin{aligned}&\hspace{.25in}\bullet \,\textrm{Vol}^{n-1}_{g_k}(\partial \Omega _{k,\eta })\leqslant \Psi (\eta ).&\end{aligned}$$

In particular, $$|\textrm{Vol}^n_{g_k}(\mathbb {S}^n)-\textrm{Vol}^n_{g_0^n}(\mathbb {S}^n)|\leqslant \Psi (k^{-1})$$.

### Proof

Clearly the full volume convergence follows from (5.13) and (5.14) by taking $\eta >0$ arbitrarily small. (5.13) and (5.15) are implied directly by the $C^0$ Cheeger–Gromov convergence of Lemma 5.7, so it just remains to establish (5.14). To do so, we recall (2.7) for the volume tensor

$$\begin{aligned} d\textrm{Vol}^n_{g_k}=\frac{r^{n-1}}{V_{k,-}(r)^{1/2}}d\mathcal {L}^1(r)\otimes d\textrm{Vol}^{n-1}_{g_0^{n-1}} \end{aligned}$$

valid on the open hemisphere $\mathbb {S}^n_-$, and analogously on $\mathbb {S}^n_+$. We may thus estimate

$$\begin{aligned} \textrm{Vol}^n_{g_k}\left( [0,\eta ]\times \mathbb {S}^{n-1}\right)&=n\omega _n\int _0^\eta \frac{r^{n-1}}{V_{k,-}(r)^{1/2}}dr\\&\leqslant n\omega _n\eta ^{n-1}\int _0^\eta \frac{1}{V_{k,-}(r)^{1/2}}dr\\&\leqslant n\omega _n\eta ^{n-1}D=\Psi (\eta ). \end{aligned}$$

Indeed, the integral in the penultimate line is the arc length of a segment of a meridian of $(\mathbb {S}^n, g_k)$ starting from a pole. Since every meridian gives a minimizing path from one pole to the other (while also realizing the diameter of $(\mathbb {S}^n, g_k)$—see the proof of Lemma 2.1 in [41]), we arrive at the final bound. The other component of $\mathbb {S}^n\setminus \Omega _{k,\eta }$ enjoys an analogous estimate, so we may conclude. $\square$

At last, we may prove Theorems 5.1 and 5.2.

### Proof of Theorem 5.1

We begin with a sequence of would-be counterexample metrics as in (5.9), with the property that there were some $\delta _0>0$ such that

$$\begin{aligned} \textrm{d}_{\mathcal{V}\mathcal{F}}\left( (\mathbb {S}^n, g_k),(\mathbb {S}^n, g_{0}^n)\right) \geqslant \delta _0. \end{aligned}$$

Putting together Lemmas 5.7, 5.8, 5.9, 5.10 and Theorem 2.12 yields for all small $0<\eta<<1$ and all $k\gg 1$ large:

$$\begin{aligned} \textrm{d}_{\mathcal{V}\mathcal{F}}\left( (\mathbb {S}^n, g_k),(\mathbb {S}^n, g_{0}^n)\right) \leqslant \Psi (k^{-1}, \eta ). \end{aligned}$$

Taking $\eta \searrow 0$ arbitrarily small and sending $k\rightarrow \infty$ thereby contradicts the assumption that the manifolds $(\mathbb {S}^n, g_k)$ remain bounded away from $(\mathbb {S}^n, g_{0}^n)$ in the $\mathcal{V}\mathcal{F}$ distance, establishing Theorem 5.1. $\square$

### Proof of Theorem 5.2

**Fig. 3:**
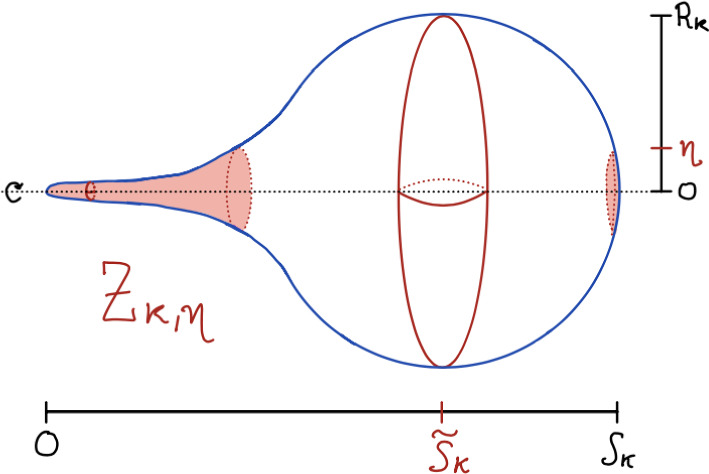
The “bad” set $Z_{k,\eta }$ which is surgically removed to obtain convergence

We begin with a sequence of would-be counterexample metrics as in (5.9), with the property that there were some $\delta _0>0$ such that for every choice of smooth $Z_k\subset \mathbb {S}^n$ with at most two connected components satisfying

$$\begin{aligned} \textrm{Vol}^n_{g_k}(Z_k)+\textrm{Vol}^{n-1}_{g_k}(\partial Z_k)\leqslant \delta _0, \end{aligned}$$

it is nonetheless true that $\textrm{d}_{\textrm{GH}}((\mathbb {S}^n\setminus Z_k, g_k), (\mathbb {S}^n, g_{0}^n))\geqslant \delta _0$. Set $Z_{k,\eta }=\mathbb {S}^n\setminus \overline{\Omega _{k,\eta }}$ (see Fig. 3).

Since

$$\begin{aligned} \textrm{d}_{\textrm{H}}\left( (\mathbb {S}^n\setminus \overline{\Omega _{{g_0^n},\eta }}, g_{0}^n),(\mathbb {S}^n, g_{0}^n)\right) \leqslant \Psi (\eta ), \end{aligned}$$

Lakzian–Sormani’s Theorem 2.12 together with Lemmas 5.7, 5.8, 5.9, 5.10 yield as before

$$\begin{aligned}&\textrm{d}_{\textrm{GH}}\left( (\mathbb {S}^n\setminus Z_{k,\eta }, g_k),(\mathbb {S}^n, g_{0}^n)\right) \leqslant \Psi (k^{-1}, \eta )\\&\textrm{Vol}^n_{g_k}(Z_{k,\eta })+\textrm{Vol}_{g_k}^{n-1}(\partial Z_{k,\eta })\leqslant \Psi (\eta : k^{-1}). \end{aligned}$$

Taking $\eta \searrow 0$ arbitrarily small and sending $k\rightarrow \infty$ thereby contradicts the assumption that the manifolds $(\mathbb {S}^n\setminus Z_{k,\eta }, g_k)$ must remain bounded away from $(\mathbb {S}^n, g_{0}^n)$ in the Gromov–Hausdorff distance, establishing Theorem 5.2. $\square$

## Stability of the Width: Proof of Theorem C

In this section, we prove Theorem C. To that end we consider rotationally symmetric metrics on the *n*-sphere $\mathbb {S}^n$, $n\geqslant 3$, satisfying:

6.1 $$\begin{aligned}&\hspace{.25in}\bullet \,\textrm{Scal}_g\geqslant n(n-1)(1-\varepsilon )^2,&\end{aligned}$$

6.2 $$\begin{aligned}&\hspace{.25in}\bullet \,\textrm{Ric}_g\geqslant 0,&\end{aligned}$$

6.3 $$\begin{aligned}&\hspace{.25in}\bullet \,\textbf{W}^n_g\geqslant \omega _{n-1}(1-\varepsilon )^{n-1}, \text { where } W^3_g =W_g \text { and, for } n\geqslant 4, W^n_g=W^{\textrm{rot}}_g.&\end{aligned}$$

By imposing the condition that *g* has non-negative Ricci curvature, we obtain measured Gromov–Hausdorff stability without the assumed diameter bound and without the removal of the “bad” set using the proof of Theorem 5.1 and Proposition 4.1. In this situation, we can also phrase the $\textrm{MinA}_g$ condition in terms of $W_g$ in dimension 3 and $W^{\textrm{rot}}_g$ in dimensions at least 4 instead, by Lemma 4.4. Indeed, all that is important in the proof of Theorem 5.1 is that no leaf of the sweepout $\Sigma _s$ is minimal if $s\ne 0$. Thus we obtain the following (a restatement of Theorem C), which is another stabilized version of Marques–Neves’ Theorem 2.3 in general *n*-dimensions under the assumption of rotational symmetry:

### Theorem 6.1

Fix $n\geqslant 3$ and $\delta >0$. There exists an $\varepsilon =\varepsilon (n, \delta )>0$ such that if *g* is a rotationally symmetric metric on the *n*-sphere $\mathbb {S}^n$ satisfying (6.1), (6.2), and (6.3), then $\textrm{d}_{\textrm{mGH}}((\mathbb {S}^n, g), (\mathbb {S}^n, g_{0}^n))\leqslant \delta$.

As in Sect. 5, we prove Theorem 6.1 by contradiction; consider *any* sequence of smooth, rotationally symmetric metrics

6.4 $$\begin{aligned} g_k=ds^2+f_k(s)^2g_0^{n-1}\text { on } [0,S_k]\times \mathbb {S}^{n-1} \text { for } k=1, 2, \ldots \end{aligned}$$

satisfying:

6.5 $$\begin{aligned}&\hspace{.25in}\bullet \,\textrm{Scal}_{g_k}\geqslant n(n-1)(1-k^{-1})^2,&\end{aligned}$$

6.6 $$\begin{aligned}&\hspace{.25in}\bullet \,\textrm{Ric}_{g_k}\geqslant 0,&\end{aligned}$$

6.7 $$\begin{aligned}&\hspace{.25in}\bullet \,{W}^n_{g_k}\geqslant \omega _{n-1}(1-k^{-1})^{n-1}, \text { where } W^3_{g_k} =W_{g_k} \text { and, for } n\geqslant 4, W^n_{g_k}=W^\textrm{rot}_{g_k}.&\end{aligned}$$

The assumption (6.6) and Proposition 4.1 imply that there exists $D=D(n,k)$ such that

6.8 $$\begin{aligned} {\textrm{diam}}_{g_k}({\mathbb {S}}^n) \le D. \end{aligned}$$

Moreover, by using Lemma 4.4 in place of Lemma 2.8, we can divide $({\mathbb {S}}^n, g_k)$, for $k\gg 1$, into two pieces $({\mathbb {S}}^n_\pm ,g_k)$ and obtain two coordinate systems, just as how we obtained (5.10) in Sect. 5. Therefore, we can use the same notation and setup from Sect. 5. Before we complete the proof of Theorem 6.1, we need the following lemma particular to this setting.

### Lemma 6.2

(Hausdorff Convergence of the Subregions) Assume (6.5), (6.6), and (6.8). For all large enough $k\geqslant 1$, we have

$$\begin{aligned} \textrm{d}_{\textrm{H}}((\Omega _{k,\eta },g_k), (\mathbb {S}^n, g_k))\leqslant \Psi (k^{-1},\eta ) \quad \text {and}\quad \textrm{d}_{\textrm{H}}((\Omega _{{g_0^n},\eta },g_{0}^n), (\mathbb {S}^n, g_{0}^n))\leqslant \Psi (\eta ). \end{aligned}$$

### Proof

Since $(\Omega _{k,\eta },g_k)\subset (\mathbb {S}^n, g_k)$, it suffices to show that a $\Psi (k^{-1},\eta )$ open neighborhood of $(\Omega _{k,\eta },g_k)$ in $(\mathbb {S}^n, g_k)$ contains all of $(\mathbb {S}^n, g_k)$.

To show this, we consider $(s,\theta )$ coordinates for the complement of $\Omega _{k,\eta }$, where we recall that *s* is the $g_k$ distance from the pole at $s=0$ to the point with coordinates $(s,\theta )$. Without loss of generality, let us show the estimate for the connected component of $\mathbb {S}^n\setminus \Omega _{k,\eta }$ written in coordinates as

$$\begin{aligned} \left( [0,s_k]\times \mathbb {S}^{n-1}, g_k=ds^2+f_k(s)^2g_0^{n-1}\right) , \end{aligned}$$

where $s_k$ is the unique parameter less than $\tilde{S}_k$ where $\eta =f_k(s_k)$. Since $f_k(0)=0$, $f_k(s_k)=\eta$, $f_k''(s)\leqslant 0$ on $[0, s_k]$, and $f_k'(s_k)=V_k(\eta )^{1/2}\rightarrow V_{0}(\eta )^{1/2}=\sqrt{1-\eta ^2}>0$, we see directly from integration that

$$\begin{aligned} \eta =f_k(s_k)=\int _0^{s_k} f_k'(\xi )d\xi \geqslant s_kf_k'(s_k)\geqslant s_k\left( \sqrt{1-\eta ^2}-\Psi (k^{-1})\right) \end{aligned}$$

for all large enough $k\gg 1$. Therefore, $0<s_k\leqslant \Psi (\eta : k^{-1})$, telling us that the diameter of each connected component of the “missed” region $\mathbb {S}^n\setminus \Omega _{k,\eta }$ can be made arbitrarily small by taking $k\gg 1$ large enough and sending $\eta \searrow 0$. The first Hausdorff distance estimate in the Lemma thus follows.

Lastly, the second estimate follows by inspection, since the complement of $\Omega _{g_0^n, \eta }$ in the round sphere consists of two geodesic disks of radius $\Psi (\eta )$. $\square$

### Proof of Theorem 6.1

We begin with a sequence of would-be counterexample metrics as in (6.4), with the property that there were some $\delta _0>0$ such that

$$\begin{aligned} \textrm{d}_{\textrm{mGH}}\left( (\mathbb {S}^n, g_k),(\mathbb {S}^n, g_{0}^n)\right) \geqslant \delta _0. \end{aligned}$$

Putting together Lemmas 6.2, 5.7, 5.8, 5.9, 5.10 and Theorem 2.12 yields for all all $k\gg 1$ large:

$$\begin{aligned} \textrm{d}_{\textrm{mGH}}\left( (\mathbb {S}^n, g_k),(\mathbb {S}^n, g_{0}^n)\right) \leqslant \Psi (k^{-1}). \end{aligned}$$

Sending $k\rightarrow \infty$ thereby contradicts the assumption that the manifolds $(\mathbb {S}^n, g_k)$ remain bounded away from $(\mathbb {S}^n, g_{0}^n)$ in the Gromov–Hausdorff distance, establishing Theorem 6.1. $\square$

## Data Availability

Not applicable, as the work does not involve any data.

## Footnotes

1 For this reason, we restricted all of our statements to $n\geqslant 3$, even though our proof methods would also work when $n=2$.

2 Recall that the *Morse index* of a two-sided minimal hypersurface $\Sigma ^{n-1}$ in $(M^n,g)$ is the number of negative eigenvalues (counted with multiplicity) of the Jacobi operator $L^\Sigma =\Delta ^\Sigma +\textrm{Ric}_g(\nu ,\nu )+|A_\Sigma |^2$, where $\Delta ^\Sigma =\textrm{div}^\Sigma \circ \nabla ^\Sigma$, and $\nu$ is a unit normal to $\Sigma$.
